# Hydraulic Performance Modeling of Inclined Double Cutoff Walls Beneath Hydraulic Structures Using Optimized Ensemble Machine Learning

**DOI:** 10.1038/s41598-025-10990-3

**Published:** 2025-07-29

**Authors:** Mohamed Kamel Elshaarawy, Martina Zeleňáková, Asaad M. Armanuos

**Affiliations:** 1 Civil Engineering Department, Faculty of Engineering, Horus University-Egypt, New Damietta, 34517 Egypt; 2 Faculty of Civil Engineering, Institute of Environmental Engineering, Technical University of Košice, 04200 Kosice, Slovakia; 3 Irrigation and Hydraulics Engineering Department, Faculty of Engineering, Tanta University, Tanta, 31733 Egypt

**Keywords:** Hydraulic structure design, Cutoff walls, Machine learning models, Uplift force control, Seepage discharge prediction, Feature importance analysis, Computer science, Engineering, Civil engineering

## Abstract

This study investigates the effectiveness of inclined double cutoff walls installed beneath hydraulic structures by employing five machine learning models: Random Forest (RF), Adaptive Boosting (AdaBoost), eXtreme Gradient Boosting (XGBoost), Light Gradient Boosting Machine (LightGBM), and Categorical Boosting (CatBoost). A comprehensive dataset of 630 samples was gathered from previous studies, including key input variables such as the relative distance between the cutoff wall and the structure’s apron width (*L*/*B*), the inclination angle ratio between downstream and upstream cutoffs (*θ*_2_/*θ*_1_), the depth ratio of downstream to upstream cutoff walls (*d*_2_/*d*_1_), and the relative downstream cutoff depth to the permeable layer depth (*d*_2_/*D*). Outputs considered were the relative uplift force (*U*/*U*_*o*_), the relative exit hydraulic gradient (*i*_*R*_/*i*_*Ro*_), and the relative seepage discharge per unit structure length (*q*/*q*_*o*_). The dataset was split with a 70:30 ratio for training and testing. Hyperparameter optimization was conducted using Bayesian Optimization (BO) coupled with five-fold cross-validation to enhance model performance. Results showed that the CatBoost model demonstrated superior performance over other models, consistently yielding high R^2^ values, specifically surpassing 0.95, 0.93, and 0.97 for *U*/*U*_*o*_, *i*_*R*_/*i*_*Ro*_, and *q*/*q*_*o*_, respectively, along with low RMSE scores below 0.022, 0.089, and 0.019 for the same variables. A feature importance analysis is conducted using SHapley Additive exPlanations (SHAP) and Partial Dependence Plot (PDP). The analysis revealed that *L*/*B* was the most influential predictor for *U*/*U*_*o*_ and *i*_*R*_/*i*_*Ro*_, while *d*_2_/*D* played a crucial role in determining *q*/*q*_*o*_. Moreover, PDPs illustrated a positive linear relationship between *L*/*B* and *U*/*U*_*o*_, a V-shaped impact of *d*_2_/*d*_1_ on *i*_*R*_/*i*_*Ro*_ and *q*/*q*_*o*_, and complex nonlinear interactions for *θ*_2_/*θ*_1_ across all target variables. Furthermore, an interactive Graphical User Interface (GUI) was developed, enabling engineers to efficiently predict output variables and apply model insights in practical scenarios.

## Introduction

Hydraulic structures often experience seepage due to variations in hydraulic head between the upstream and downstream boundaries^1–5^. The impact of seepage discharge on the foundations of these structures includes factors such as uplift force, exit hydraulic gradient, and seepage discharge^6^. One of the earliest empirical approaches to managing seepage-related forces was the creep length hypothesis, developed by Fell^7^. This method aimed to regulate the uplift force and exit hydraulic gradient in hydraulic structures. Lane^8^ identified a substantial difference in seepage behavior along horizontal and vertical creep paths after analyzing over 200 failed hydraulic structures. The findings led to the introduction of the weighted creep theory, which accounts for seepage flow beneath hydraulic structures by assigning weighted parameters to both horizontal and vertical percolation paths^9,10^.

Further advancements in seepage analysis were introduced by Al-Juboori and Datta^11^, who formulated a mathematical approach using complex numbers to determine uplift force and exit gradient in hydraulic structure foundations. Malhotra^12^ later examined seepage conditions involving two cutoff walls of equal depth positioned at the upstream and downstream sections of an apron. Polubarinova-Kochina^13^ contributed analytical solutions for different finite seepage depth conditions, addressing cases such as flat foundations with a single cutoff wall and depressed floors without any cutoff walls. More recently, Mansuri et al.^14^ explored the effect of cutoff walls on uplift force using SEEP/W, evaluating various positions and inclinations of these walls. Their findings revealed that constructing the cutoff wall at an upstream location significantly reduced uplift force. Additionally, placing the cutoff wall at the upstream or downstream heel minimized seepage flow, whereas a centrally positioned cutoff wall resulted in the highest seepage outflow. Khalili Shayan and Amiri-Tokaldan^15^ also utilized SEEP/W software to analyze how an inclined cutoff wall influences seepage discharge, uplift force, and the exit hydraulic gradient. Their study highlighted that the optimal cutoff wall angle depends largely on its position and length. They further observed that installing a cutoff wall at the far end of the downstream section effectively reduces the exit hydraulic gradient.

Armanyous et al.^16^ employed a symmetrical physical sand box model to investigate the influence of double sheet pile depths, distance among them, and pollutant distances upstream within the sheet piles. The regionally contaminated porous environment is numerically investigated utilizing modeling software MODFLOW and M3DMS. The mean absolute variation in time comparing experimental and numerical outcomes for contamination moving through upstream to the site underneath the second sheet pile and arriving at the soil surface downstream is 9.31 and 6.11%, correspondingly. Praveenkumar et al.^17^ employed experimental simulation for investigating the impact of different cut-off angles upon the exit gradient as well as uplift pressure of hydraulic structures. The angle has been determined between floors to upstream. The outcomes revealed that putting a cutoff wall at either the upstream or downstream ends resulted in a raise in uplift head through enhancing the cutoff wall inclination. However, in an identical situation, the seepage flow reduced while the inclination increased above 90 degrees. Utilizing a downstream cutoff wall having a greater than 90-degree angle reduced exit gradients.

Salmasi et al.^18,19^ found that deeper downstream cutoff walls increase uplift force, while cutoff walls at both edges eliminate the exit gradient and reduce seepage. Sartipi et al.^20^ showed that cutoff walls can span the entire seepage path, and Zainal^3^ identified optimal inclination angles for minimizing seepage (60°), exit gradient (120–135°), and uplift force (45–75°). Armanuos et al.^6^ used Finite Element Modeling (FEM) analysis to confirm that deeper downstream cutoff walls and wider spacing lower the exit gradient, while right-angled cutoff walls of equal depth are the most effective for reducing seepage. Arshad et al.^21^ conducted a finite element analysis of seepage in Hub Dam using the SEEP/W model. They found that the original cutoff wall design significantly minimized seepage, with a flow rate of 2.2117 × 10^−4^ ft^3^/sec and an exit gradient of 0.099, making further extensions economically unjustified. Parsaie et al.^10^ studied numerically and experimentally to optimize the best location of drainage and cutoff wall small concrete dams. They demonstrated that placing a drainage well at 0.2 *L* from the upstream face and incorporating an upstream cutoff wall reduced uplift force by up to 53%, highlighting the effectiveness of optimized seepage control measures.

Recent advancements in hydraulic engineering have introduced genetic algorithms (GAs) and artificial neural networks (ANNs) to optimize cutoff wall design and placement. Hassan^22,23^ combined FEM with genetic algorithms to determine the ideal cutoff wall position and inclination under static conditions. Similarly, Javanmard et al.^24^ used FEM and GAs to identify configurations that minimize uplift force and seepage while improving structural stability. Alrowais et al.^25^ employed a Feed-Forward Neural Network (FFNN) to assess cutoff wall efficiency, finding that upstream heel placement was most effective in reducing uplift pressure. The study highlighted that inclination angles significantly impact uplift pressure, exit hydraulic gradient, and seepage flow under both static and dynamic conditions. While a 90° inclination was optimal for reducing seepage in static settings, seismic activity sustained high seepage rates regardless of inclination. FFNN predictions showed high accuracy, with R^2^ values nearing 1.00. Haghdoost et al.^26^ examined the effects of sheet pile inclination, number, and spacing using the SEEP/W finite element model. A 105° inclination reduced seepage flow by 34.8%, while wider spacing between sheet piles (10 m) led to a 45.9% reduction. Increasing the number of sheet piles further decreased seepage, with a 50% reduction observed when six sheet piles were installed.

## Research Novelty and Contribution

Despite the critical role of hydraulic structures in water resource management, the application of advanced ML models to predict key hydraulic parameters, such as uplift force, exit hydraulic gradient, and seepage discharge in hydraulics structures with inclined double-cutoff walls remains underexplored. While conventional methods have long relied on simplified analytical approaches, there is a notable lack of research leveraging ML techniques to enhance predictive accuracy and provide deeper insights into seepage behavior. Previous studies have examined the influence of cutoff wall configurations, yet they have not fully harnessed the potential of ML models to assess their hydraulic performance comprehensively.

This study addresses these gaps by integrating interpretability tools like SHAP and PDP to elucidate the impact of cutoff wall depth, location, and inclination angle on seepage dynamics and structural stability. Furthermore, earlier investigations often lacked a systematic model evaluation framework, raising concerns about the reliability of their findings. To overcome this limitation, our research implements a rigorous validation approach, ensuring model robustness and accuracy. Beyond theoretical advancements, this study bridges the gap between complex computational models and practical engineering applications by developing a user-friendly interface^27^. This tool enables water resource professionals to efficiently apply predictive models in real-world scenarios, enhancing decision-making and design optimization for hydraulic structures with inclined double-cutoff walls.

## Materials and Methods

**Fig. 1:**
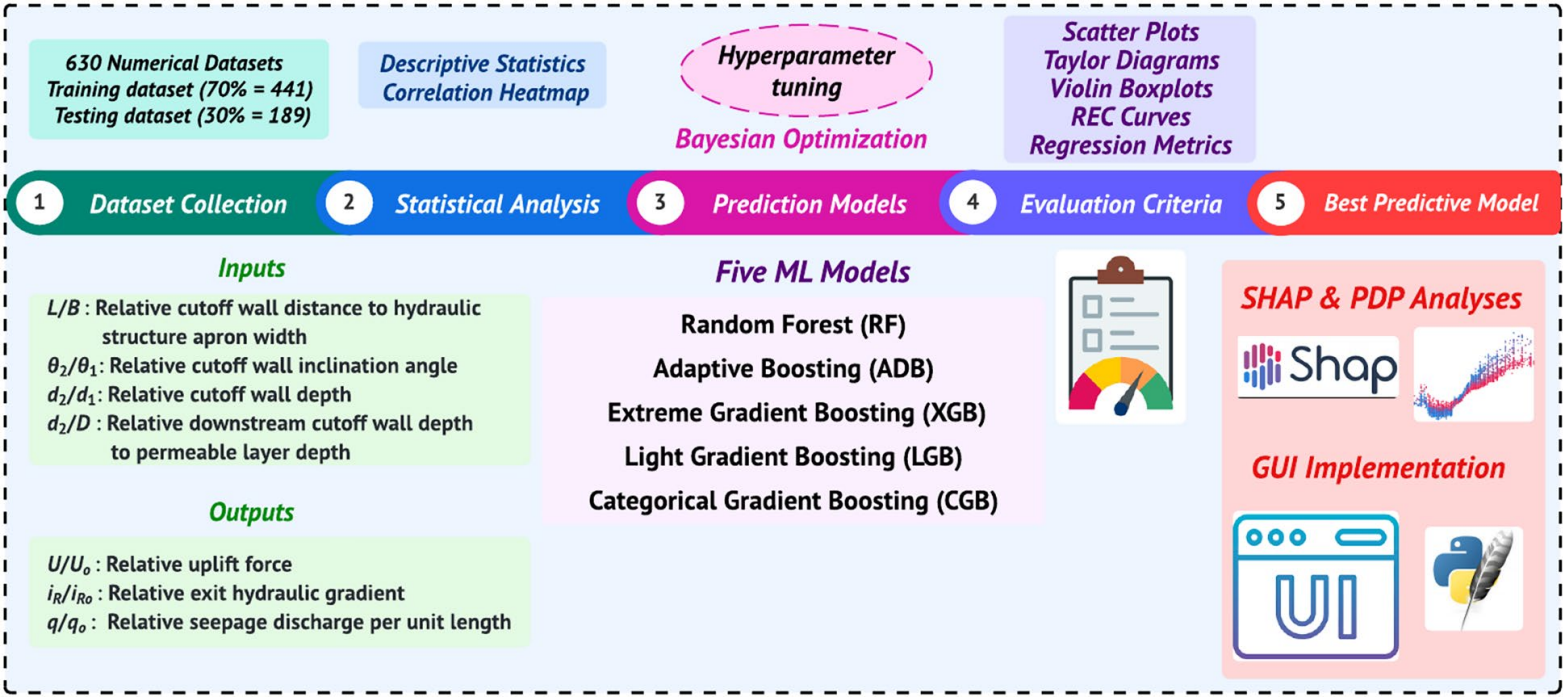
Methodology flowchart.

Figure 1 presents a comprehensive methodology for predicting flow characteristics under hydraulic structures with inclined double cutoff walls using ML models. The process begins with gathering a dataset of 630 numerical samples, followed by statistical analysis, correlation assessment, and data normalization. The proposed ML models are trained with hyperparameters optimized through BO. Model evaluation involves both visual and quantitative assessments, including regression error analysis and metrics such as the Akaike Information Criterion (AIC). In the final phase, the most effective model is selected, with SHAP and PDP used to visualize feature importance and interpretation. Additionally, a GUI is created to enable users to interact with model predictions, enhancing the accessibility and practical interpretation of results.

### Database collection

**Fig. 2:**
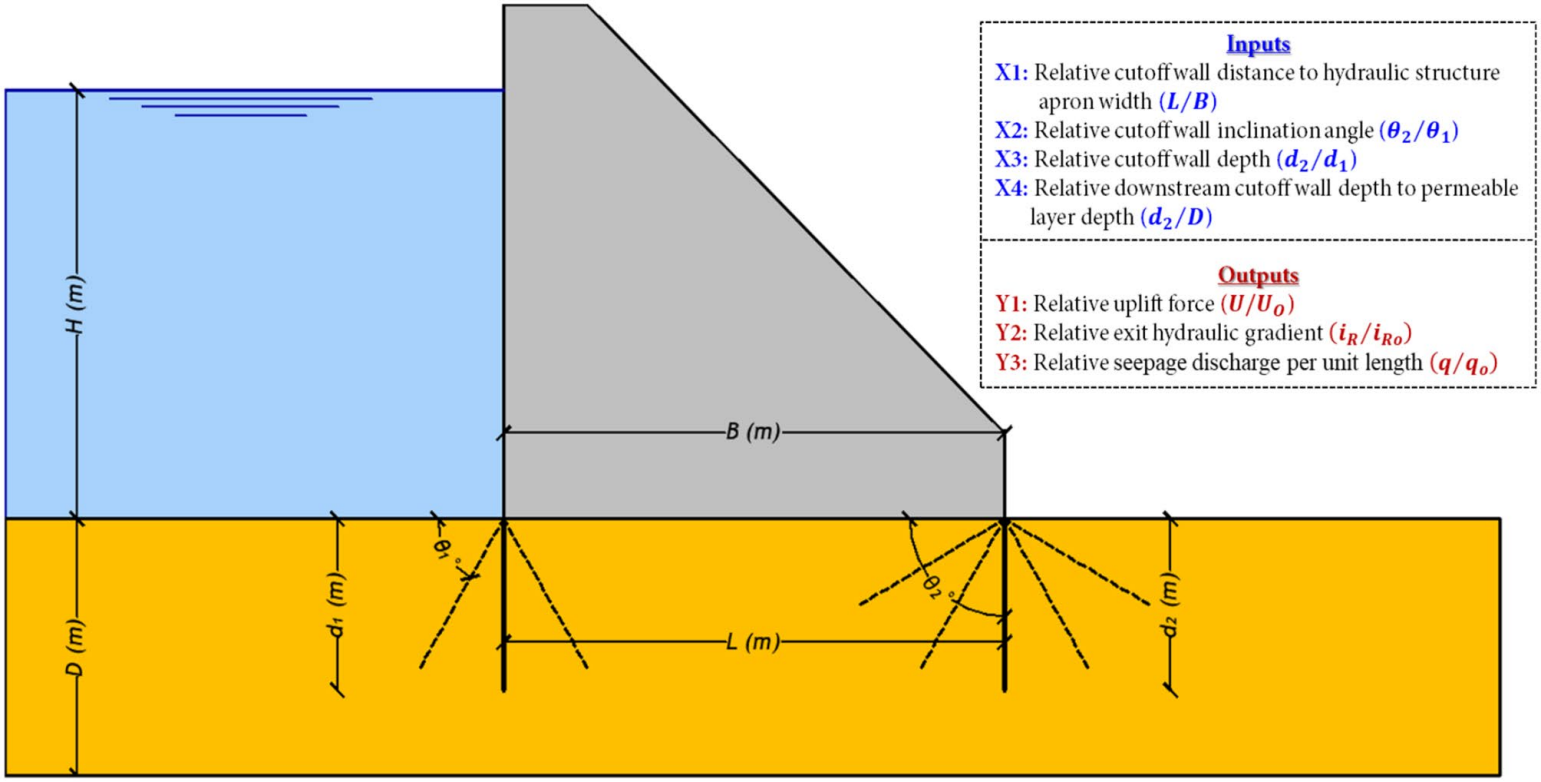
Schematic of a double inclined cutoff wall under a hydraulic structure with the investigated parameters.

A comprehensive database comprising 630 numerical scenarios was gathered from Armanuos et al.^6^ to examine the influence of inclined double-cutoff walls on key hydraulic parameters, including uplift forces, seepage discharge, and exit hydraulic gradient beneath hydraulic structures. This dataset played a crucial role in constructing the predictive models used in this study. Figure 2 illustrates a schematic representation of a hydraulic structure with inclined double-cutoff walls, along with the key parameters investigated. The study considered multiple factors, including the density of water (*ρ*) and gravitational acceleration (*g*) as fluid properties, while the hydraulic conductivity (*k*) of the porous medium represented the permeability characteristics of the foundation soil. The structural dimensions analyzed encompassed the depth of the upstream cutoff wall (*d*₁), depth of the downstream cutoff wall (*d*₂), and the total depth of the permeable layer (*D*). Additionally, the apron width of the hydraulic structure (*B*) and the distance between the upstream and downstream cutoff walls (*L*) were considered to assess their impact on seepage behavior.

Through dimensional analysis, Eq. (1) is derived as follows:

1 $$\phi \left( {\rho ,g,k,d_{1} ,d_{2} ,D, B,L,H,\theta_{1} ,\theta_{2} ,i_{R} ,i_{Ro} ,q,q_{o} ,U,U_{o} } \right) = 0$$

Applying Buckingham’s π theorem^28^ and selecting *H*, *k*, and *g* as the repeating variables, the total number of influencing variables (n) in this problem is 17, while the number of fundamental dimensions (*m*) involved is 3, namely L (length), M (mass), and T (time). This selection of variables follows the approach used by Armanuos et al.^6^ Consequently, the number of resulting dimensionless parameters (π-terms) is 14, as formulated in Eqs. (2) and (3):

2 $$\phi \left( {\pi_{1} ,\pi_{2} ,\pi_{3} ,\pi_{4} ,\pi_{5} ,\pi_{6} ,\pi_{7} ,\pi_{8} ,\pi_{9} ,\pi_{10} ,\pi_{11} ,\pi_{12} ,\pi_{13} ,\pi_{14} } \right) = 0$$

3 $$\phi \left( {\frac{q}{kH},\frac{{q_{o} }}{kH},\frac{U}{{k^{2} H\rho }},\frac{{U_{o} }}{{k^{2} H\rho }},i_{R} ,i_{Ro} ,\frac{B}{H},\frac{L}{H},\frac{{d_{1} }}{H},\frac{{d_{2} }}{H},\frac{D}{H},\frac{gH}{{k^{2} }},\theta_{1} ,\theta_{2} } \right) = 0$$

where *φ* represents the functional relationship among these variables. Notably, *k*, *H*, and *g* are treated as constants, as demonstrated in previous studies^6,9^. As a result, simplified expressions were formulated to estimate key relative hydraulic parameters, including relative uplift force (*U*/*U*_*o*_), relative exit hydraulic gradient (*i*_*R*_/*i*_*Ro*_), and relative seepage discharge per unit length (*q*/*q*_*o*_). These are expressed in Eqs. (4–6):

4 $$\frac{U}{{U_{o} }} = \phi \left( {\frac{L}{B},\frac{{\theta_{2} }}{{\theta_{1} }},\frac{{d_{2} }}{{d_{1} }},\frac{{d_{2} }}{D}} \right)$$

5 $$\frac{{i_{R} }}{{i_{Ro} }} = \phi \left( {\frac{L}{B},\frac{{\theta_{2} }}{{\theta_{1} }},\frac{{d_{2} }}{{d_{1} }},\frac{{d_{2} }}{D}} \right)$$

6 $$\frac{q}{{q_{o} }} = \phi \left( {\frac{L}{B},\frac{{\theta_{2} }}{{\theta_{1} }},\frac{{d_{2} }}{{d_{1} }},\frac{{d_{2} }}{D}} \right)$$

where *L*/*B* represents the relative cutoff wall distance to the hydraulic structure apron width, *θ*_2_/*θ*_1_ denotes the relative cutoff wall inclination angle,* d*_2_/*d*_1_ refers to the relative cutoff wall depth, and *d*_2_/*D*​​ corresponds to the relative downstream cutoff wall depth to the permeable layer depth.

### Descriptive statistics

Table 1 provides a descriptive statistics of each variable across 630 records, covering both input parameters (*L*/*B*, *θ*_2_/*θ*_1_, *d*_2_/*d*_1_, *d*_2_/*D*) and output targets (*U*/*U*_*o*_, *i*_*R*_/*i*_*Ro*_, *q*/*q*_*o*_). For *L*/*B*, values range from 0.500 to 1.000, with both the mean and median at 0.750, suggesting a balanced, symmetrical distribution centered around this value. The standard deviation (SD) of 0.250 indicates that values are closely clustered around the mean. The variable *θ*_2_/*θ*_1_ displays greater variability, with values spanning from 0.250 to 2.500. With a mean of 1.083 and a median of 1.000, there is a slight skew towards lower values. An SD-value of 0.617 shows moderate dispersion. Similarly, *d*_2_/*d*_1_ ranges from 0.500 to 2.000. The mean of 1.167 and median of 1.000 suggest a slight right skew, with an SD-value of 0.624 indicating moderate variability. The parameter *d*_2_/*D* has a narrower range, from 0.200 to 0.800, with both the mean and median at 0.500, pointing to a symmetrical distribution around this central value. The low SD-value of 0.200 reflects limited variability among data points.

**Table 1:** Statistical description of the studied input and output variables.

Parameters	Inputs				Outputs		
	*L*/*B*	*θ*_2_/*θ*_1_	*d*_2_/*d*_1_	*d*_2_/*D*	*U*/*U*_*o*_	*i*_*R*_/*i*_*Ro*_	*q*/*q*_*o*_
Min	0.500	0.250	0.500	0.200	0.436	0.000	0.422
Max	1.000	2.500	2.000	0.800	1.261	1.020	0.952
Mean	0.750	1.083	1.167	0.500	0.863	0.550	0.741
Median	0.750	1.000	1.000	0.500	0.865	0.664	0.755
SD	0.250	0.617	0.624	0.200	0.145	0.340	0.122

For the output variable *U*/*U*_*o*_, values range from 0.436 to 1.261, with a mean of 0.863 and a median of 0.865, indicating a nearly symmetric distribution and an SD-value of 0.145, which shows relatively low dispersion. *i*_*R*_/*i*_*Ro*_ has a wider range from 0.000 to 1.020, with a mean of 0.550 and a median of 0.664. The SD-value is higher at 0.340, indicating more variability. *q*/*q*_*o*_ ranges from 0.422 to 0.952, with a mean of 0.741 and a median of 0.755, showing slight skew towards lower values. Its relatively low an SD-value of 0.122 indicates close clustering around the mean. Overall, these descriptive statistics reveal that most variables exhibit symmetrical or nearly symmetrical distributions, with varying levels of dispersion. The dataset maintains uniformity with a consistent count of 630 for all variables, supporting robust model analysis and interpretation.

### *Correlation analysis*

**Fig. 3:**
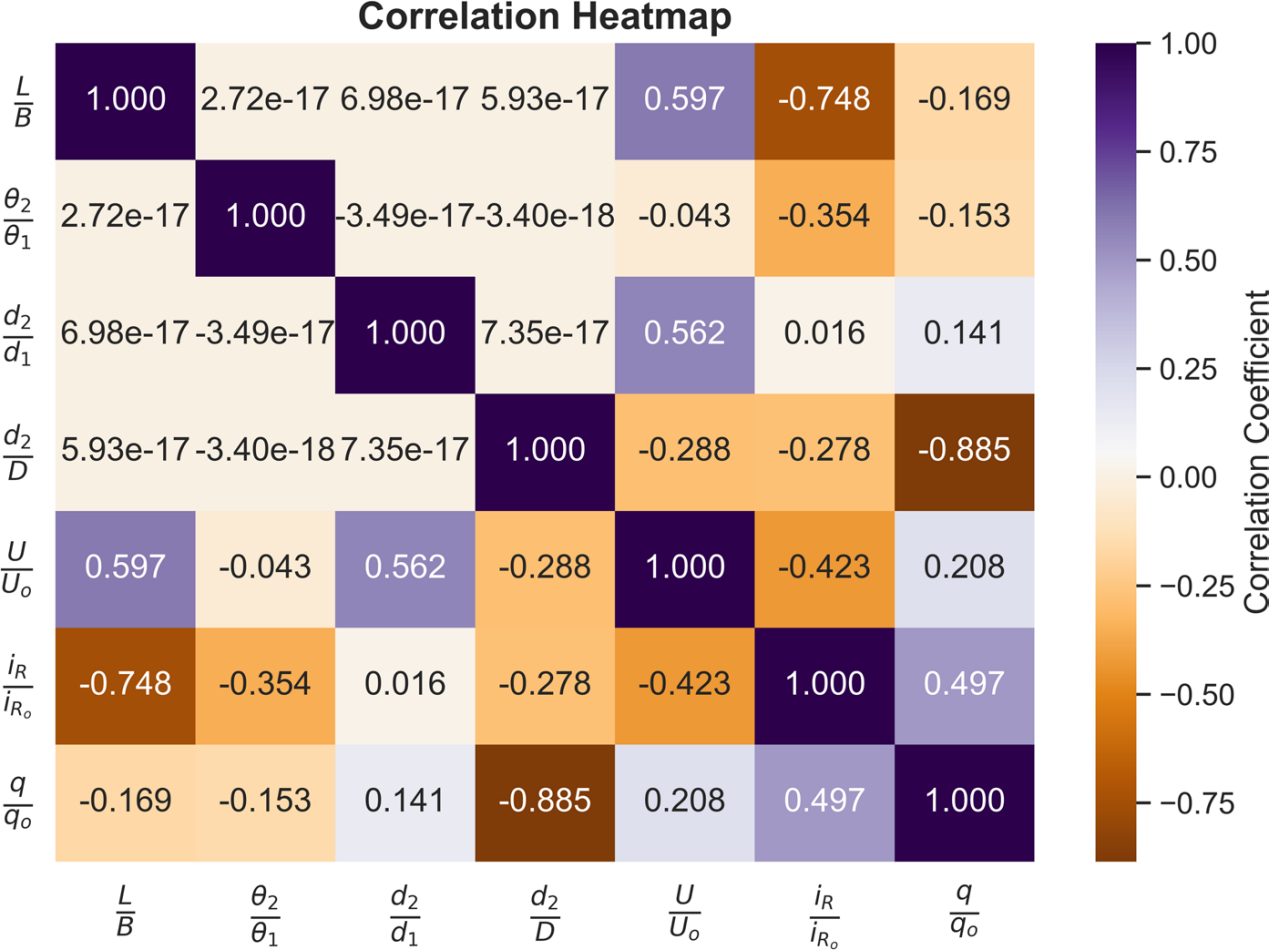
Correlation heatmap indicating the relation between variables.

Figure 3 illustrates a correlation heatmap, which visualizes the correlation matrix of the dataset’s variables. The heatmap reveals that *L*/*B* has a moderate positive correlation of 0.597 with *U*/*U*_*o*_, suggesting that as *L*/*B* increases, *U*/*U*_*o*_ also tends to rise. In contrast, *L*/*B* has negative correlations with *i*_*R*_/*i*_*Ro*_ (− 0.748) and *q*/*q*_*o*_ (− 0.169), indicating moderate to weak inverse relationships with these variables. No significant correlations are observed between *L*/*B* and *θ*_2_/*θ*_1_, *d*_2_/*d*_1_, or *d*_2_/*D*, as these values are close to zero. The variable *θ*_2_/*θ*_1_ generally shows weak to negligible correlations with other variables, with the exception of a moderate negative correlation of − 0.354 with *i*_*R*_/*i*_*Ro*_, indicating a slight inverse relationship. *d*_2_/*d*_1_ demonstrates positive associations with *U*/*U*_*o*_ (0.562) and *q*/*q*_*o*_ (0.141), implying weak to moderate positive relationships. However, *d*_2_/*d*_1_ shows virtually no correlation with *i*_*R*_/*i*_*Ro*_ (0.0155), indicating independence from this variable. Similarly, *d*_2_/*D* is weakly negatively correlated with *U*/*U*_*o*_ (− 0.288) and *i*_*R*_/*i*_*Ro*_ (− 0.278), suggesting minor inverse relationships. However, a strong negative correlation with *q*/*q*_*o*_ (− 0.885) indicates that increases in *d*_2_/*D* are associated with significant decreases in *q*/*q*_*o*_.

For *U*/*U*_*o*_, there are moderate positive correlations with *L*/*B* (0.597) and *d*_2_/*d*_1_ (0.562), showing that increases in these variables are associated with increases in *U*/*U*_*o*_. Conversely, *U*/*U*_*o*_ has a weak negative correlation with *d*_2_/*D* (− 0.288) and essentially no relationship with *θ*_2_/*θ*_1_ (− 0.0432). *U*/*U*_*o*_ also has weak to moderate inverse correlations with *i*_*R*_/*i*_*Ro*_ (− 0.423) and a weak positive relationship with *q*/*q*_*o*_ (0.208). The variable *i*_*R*_/*i*_*Ro*_ has moderate to weak negative correlations with *L*/*B* (− 0.748), *θ*_2_/*θ*_1_ (− 0.354), and *d*_2_/*D* (− 0.278), indicating inverse relationships. Its correlation with *U*/*U*_*o*_ is − 0.423, also a weak inverse relationship. However, *i*_*R*_/*i*_*Ro*_ shares a moderate positive correlation of 0.497 with *q*/*q*_*o*_, suggesting that these variables tend to increase together. Finally, *q*/*q*_*o*_ is strongly negatively correlated with *d*_2_/*D* (− 0.885), indicating a robust inverse association. It shows weaker negative correlations with *L*/*B* (− 0.169) and *θ*_2_/*θ*_1_ (− 0.153), and a weak positive correlation with *d*_2_/*d*_1_ (0.141). Among its relationships with other variables, *q*/*q*_*o*_ has a moderate positive correlation with *i*_*R*_/*i*_*Ro*_ (0.497) and a weak positive relationship with *U*/*U*_*o*_ (0.208).

### Description of ML models

In this study, the software environment used for model development and implementation was Anaconda3 2024.10-1 (Python 3.12.7 64-bit), distributed by Anaconda, Inc. Anaconda can be accessed and downloaded from: https://www.anaconda.com. The ML models adopted in this study to predict *U*/*U*_*o*_, *i*_*R*_/*i*_*Ro*_, and *q*/*q*_*o*_ are RF, AdaBoost, XGBoost, LightGBM, and CatBoost. RF was selected for its robustness in handling large datasets and its ability to model complex relationships by constructing multiple decision trees^29^. This ensemble method helps mitigate overfitting through bootstrapping and aggregation, making it ideal for tasks involving non-linear dependencies. The Scikit-learn library was used to implement the RF model, allowing for efficient training and evaluation. The AdaBoost model was chosen for its simplicity and effectiveness in improving the performance of weak learners, particularly decision trees^30^. By iteratively focusing on difficult-to-classify samples, it is able to correct errors from previous learners, making it well-suited for regression tasks where outliers or hard-to-predict data points exist. This method was implemented using the Scikit-learn library, leveraging its built-in functionality for boosting algorithms^31^.

The XGBoost model was selected for its speed, scalability, and superior performance in many ML competitions. This gradient boosting method incorporates regularization techniques that reduce overfitting and improve generalization, making it particularly effective for tasks involving large datasets and complex, non-linear relationships. The XGBoost library was used to implement this model, which is known for its efficiency and predictive accuracy. The LightGBM model was chosen due to its ability to efficiently handle large datasets, faster training time, and its capability to handle categorical variables without extensive preprocessing^32^. This model uses histogram-based algorithms to increase computational efficiency and scalability, making it particularly well-suited for large-scale regression tasks. The LightGBM library was used to implement this model, benefiting from its optimized performance for handling large-scale data. The CatBoost model was selected for its ability to handle both categorical and numerical data without the need for extensive preprocessing, which makes it particularly well-suited for real-world data that may contain a mix of feature types^33^. This gradient boosting model is designed to improve training speed and accuracy, especially in cases where feature interactions are important. The CatBoost library was used to implement this model, taking advantage of its powerful handling of categorical variables and its excellent performance on structured datasets^34^.

The RF model is an ensemble ML algorithm that generates multiple decision trees through a process called bootstrap aggregation, commonly known as bagging^35^. In this approach, each tree is trained on a different random subset of the data selected with replacement, which introduces variation among the trees. Approximately two-thirds of the dataset is used to train each tree, while the remaining one-third, known as out-of-bag (OOB) data, is utilized for internal validation and estimating prediction error. For regression problems, the final prediction is computed as the average output of all individual trees. In this model (Eq. 7), *T* represents the total number of trees, and ${\widehat{y}}^{\left(t\right)}$ denotes the prediction generated by the *t*-th tree^36^.

The AdaBoost model is another ensemble strategy that transforms a set of weak learners into a strong predictive model through sequential training^37^. Each successive model focuses more on the training instances that were incorrectly classified by its predecessors. Initially, equal weights are assigned to all instances, but these weights are adjusted after each iteration to give more emphasis to difficult cases. The significance of each weak learner in the final model is determined by its accuracy, with more accurate learners receiving higher weights. In this formulation (Eq. 8), ${h}_{t}\left(x\right)$ refers to the output of the *t*-th weak learner, and ${\alpha }_{t}$ represents the associated weight based on performance^38^. The XGBoost model enhances traditional gradient boosting by incorporating additional features such as regularization, efficient handling of missing data, and parallel processing capabilities^39^. It builds an additive model in a stage-wise manner by minimizing a loss function using gradient descent. At each step, a new regression tree is trained to approximate the negative gradient of the loss function and is added to the existing ensemble. In this model (Eq. 9), $\eta$ denotes the learning rate, and ${f}_{t}\left(x\right)$ is the regression tree added at iteration *t*^40^.

The LightGBM model is a high-efficiency gradient boosting framework that uses a histogram-based approach and grows trees leaf-wise instead of level-wise^41–43^. This strategy often results in faster training times and improved accuracy, especially when applied to large-scale datasets. The model output is obtained by summing the predictions of all trees in the ensemble (Eq. 10), where ${f}_{k}\left(x\right)$ indicates the prediction from the *k*-th individual tree. The CatBoost model is a gradient boosting algorithm optimized for handling both numerical and categorical variables with minimal preprocessing. It employs ordered boosting to prevent overfitting and data leakage and builds symmetric decision trees to ensure computational consistency and efficiency^44,45^. The final prediction is formed by aggregating the weighted outputs of each tree in the model. In Eq. (11), ${\theta }_{k}$​ represents the weight of the *k*-th tree, and ${f}_{k}\left(x\right)$ is its corresponding output.

7 $$\hat{y} = \frac{1}{T}\mathop \sum \limits_{t = 1}^{T} \hat{y}^{\left( t \right)}$$

8 $$\hat{y} = \mathop \sum \limits_{t = 1}^{T} \alpha_{t} h_{t} \left( x \right)$$

9 $$\hat{y}_{t} = \hat{y}_{t - 1} + \eta \cdot f_{t} \left( x \right)$$

10 $$\hat{y} = \mathop \sum \limits_{k = 1}^{K} f_{k} \left( x \right)$$

11 $$\hat{y} = \mathop \sum \limits_{k = 1}^{K} \theta_{k} f_{k} \left( x \right)$$

### Hyperparameters tuning

Optimizing hyperparameters is essential to improve model accuracy and reliability^46–53^. While grid search and random search are frequently used methods, they often require extensive computation time and may yield inconsistent results. BO, on the other hand, is more efficient, as it builds on prior search results to identify optimal solutions more quickly^54^. This study applies BO in combination with five-fold cross-validation (BO + 5CV) to optimize model tuning while reducing overfitting^55^. The fivefold CV strikes a balance between computational efficiency and evaluation quality, requiring fewer iterations than higher fold values, such as 10, yet still providing reliable performance assessments as demonstrated in Refs^48,57^.. This approach makes BO + 5CV a robust and effective choice for hyperparameter tuning.

### Evaluation criteria

The performance evaluation of predictive models is vital for establishing scientific validity and practical effectiveness^58–60^. While training datasets help fit models to existing data, testing datasets are essential to assess generalization to new data, ensuring the model’s robustness and applicability^61^. This study employs visual and quantitative methods for model evaluation. Visual assessments, like scatter plots in regression, intuitively display relationships and outliers. Quantitative assessments use metrics such as AIC, uncertainty analysis, and REC curves to objectively compare models, aiding in selecting the best model for regression tasks. Together, these methods ensure both scientific reliability and practical relevance^62^.

### Regression indices

To rigorously assess the predictive performance of regression models, various indices are employed to capture distinct aspects of model accuracy and reliability^63^. Table 2 presents the mathematical formulations for the metrics used, offering a structured approach to model evaluation^64^. Where, *n* represents the total number of data points in the dataset; ${y}_{i}$ and $\widehat{{y}_{i}}$ denote the actual and predicted values for the *i*-th data point, respectively; $\overline{y }$ is the mean of the actual values, and $\overline{\widehat{y} }$ is the mean of the predicted values^65^. Together, these metrics provide a comprehensive picture of model performance, evaluating elements such as goodness of fit, bias, and error magnitude, which are crucial for comparing models and identifying the most precise and dependable option for engineering applications^66^.

**Table 2:** Statistical indicators used to evaluate and compare the predictive performance of the ML models.

Metric	Formula
Determination coefficient	${\text{R}}^{2} = 1 - \frac{{\mathop \sum \nolimits_{i = 1}^{n} \left( {y_{i} - \widehat{{y_{i} }}} \right)^{2} }}{{\mathop \sum \nolimits_{i = 1}^{n} \left( {y_{{i{ }}} - \overline{y}} \right)^{2} }}$
Root mean squared error	${\text{RMSE}} = \sqrt {\frac{{\mathop \sum \nolimits_{i = 1}^{{{ }n}} \left( {y_{i} - \widehat{{y_{i} }}} \right)^{2} }}{n}{ }}$
Root mean squared relative error	${\text{RMSRE}} = \sqrt {\frac{1}{n}\mathop \sum \limits_{i = 1}^{{{ }n}} \left( {\frac{{y_{i} - \widehat{{y_{i} }}}}{{y_{i} }}} \right)^{2} }$
Mean absolute error	${\text{MAE}} = \frac{{\mathop \sum \nolimits_{i = 1}^{{{ }n}} \left| {y_{i} - \widehat{{y_{i} }}} \right|}}{n}$
Mean absolute relative error	${\text{MARE}} = \frac{{\mathop \sum \nolimits_{i = 1}^{{{ }n}} \left| {\frac{{y_{i} - \widehat{{y_{i} }}}}{{y_{i} }}} \right|}}{n}$
Percent bias	${\text{PBIAS}} = \frac{{\mathop \sum \nolimits_{i = 1}^{{{ }n}} \left( {\widehat{{y_{i} }} - y_{i} } \right)}}{{\mathop \sum \nolimits_{i = 1}^{{{ }n}} \left( {y_{i} } \right)}} \times 100$

### Model complexity

A further statistical tool, the Akaike Information Criterion (AIC), developed by Hirotsugu Akaike in the 1970s, serves as a vital measure for model selection by balancing model complexity with fit accuracy^67^. It can be calculated using the following formula (Eq. 12), where RSS the residual sum of squares and *K* is the number of parameters.

12 $${\text{AIC}} = 2K + n\log \left( {\frac{{{\text{RSS}}}}{n}} \right)$$

AIC estimates the information loss when a model approximates the true data-generating process. A lower AIC value generally indicates a more desirable model, striking a balance between simplicity and accuracy and reducing the likelihood of overfitting or underfitting^68^.

### Uncertainty analysis

Uncertainty analysis evaluates the reliability of model predictions by quantifying uncertainty arising from experimental conditions, input variations, and model outputs^69^. This study employs the *U*_95_ formula (Eq. 13), which calculates a 95% confidence interval based on RMSE and standard deviation, ensuring robust interpretation of predictive accuracy. By analyzing *U*_95_ values, the study provides deeper insights into model reliability, enabling informed decision-making and improving confidence in machine learning-based predictions^70^.

13 $${\text{U}}_{{{95}}} = {1}.{96}\sqrt {{\text{RMSE}}^{2} + {\text{SD}}^{2} }$$

### Model interpretability

SHAP and PDPs are powerful tools for understanding feature importance in machine learning models. SHAP values, rooted in cooperative game theory, fairly distribute prediction contributions among input features by calculating their marginal impacts, providing both global and local interpretability^71–74^. This ensures transparency in complex models by explaining individual predictions. PDPs, on the other hand, visualize how a feature influences model predictions by averaging out other effects, making them useful for assessing global feature importance^75,76^. While PDPs help reveal general trends, they assume feature independence, which may limit their effectiveness when features are highly correlated^77^.

### Model deployment

Integrating machine learning models into desktop applications enhances their accessibility and practical usability. Applications developed using frameworks such as Tkinter^78^ enable end-users to interact with predictive models directly on their personal computers, offering advantages such as quick response times and offline functionality. Embedding an ML model within a Tkinter-based graphical user interface (GUI) allows users to input data and receive predictions in a seamless manner. This approach is particularly beneficial for non-technical users, as it simplifies the use of complex algorithms through a user-friendly interface^72,80^.

## Results

### Model optimization

Table 3 outlines a comparative analysis of the ML models’ performance before and after hyperparameter optimization based on the RMSE metric. Prior to tuning, the CatBoost algorithm exhibited the best performance for the *U*/*U*_*o*_ output, achieving the lowest RMSE on both datasets, which reflects its strong initial accuracy. Post-optimization, all models showed a modest rise in RMSE on the training data which is an anticipated outcome that typically signifies enhanced generalization and reduced risk of overfitting. Notably, the CatBoost model maintained its edge post-tuning with a test RMSE of 0.0288. The LightGBM and XGBoost models also showed competitive performance post-tuning, with test RMSE values of 0.0298 and 0.0313, respectively.

**Table 3:** Comparison of model performance before and after hyperparameter tuning based on RMSE for training and testing datasets.

Output	Model	Best hyperparemeters	Pre-tuning		Post-tuning	
			Train RMSE	Test RMSE	Train RMSE	Test RMSE
*U*/*U*_*o*_	RF	n_estimators = 1000; max_depth = 30; min_samples_split = 6; min_samples_leaf = 3	0.0515	0.0247	0.0552	0.0330
	AdaBoost	n_estimators = 275; learning_rate = 0.866; loss = square	0.0519	0.0466	0.0552	0.0476
	XGBoost	n_estimators = 1000; learning_rate = 0.0303; max_depth = 30; min_child_weight = 9; subsample = 0.364; colsample_bytree = 1	0.0255	0.0214	0.0321	0.0313
	LightGBM	n_estimators = 387; learning_rate = 0.252; max_depth = 26; num_leaves = 23; min_child_samples = 42; subsample = 0.1; colsample_bytree = 0.959	0.0240	0.0225	0.0327	0.0298
	CatBoost	depth = 3; learning_rate = 0.0898; l2_leaf_reg = 1	0.0203	0.0212	0.0292	0.0288
*i*_*R*_/*i*_*Ro*_	RF	n_estimators = 255; max_depth = 17; min_samples_split = 2; min_samples_leaf = 4	0.1047	0.0724	0.1156	0.0979
	AdaBoost	n_estimators = 1000; learning_rate = 0.0204; loss = exponential	0.1072	0.0989	0.1199	0.1038
	XGBoost	n_estimators = 407; learning_rate = 0.0169; max_depth = 5; min_child_weight = 11; subsample = 1; colsample_bytree = 1	0.0749	0.0664	0.0947	0.0959
	LightGBM	n_estimators = 387; learning_rate = 0.252; max_depth = 26; num_leaves = 23; min_child_samples = 42; subsample = 0.1; colsample_bytree = 0.959	0.0695	0.0673	0.0956	0.0958
	CatBoost	depth = 3; learning_rate = 0.3; l2_leaf_reg = 9.515	0.0643	0.0688	0.0927	0.0884
*q*/*q*_*o*_	RF	n_estimators = 526; max_depth = 30; min_samples_split = 4; min_samples_leaf = 1	0.0485	0.0179	0.0493	0.0339
	AdaBoost	n_estimators = 1000; learning_rate = 0.9679; loss = square	0.0507	0.0358	0.0513	0.0366
	XGBoost	n_estimators = 905; learning_rate = 0.0752; max_depth = 16; min_child_weight = 15; subsample = 0.745; colsample_bytree = 0.8196	0.0183	0.0131	0.0201	0.0198
	LightGBM	n_estimators = 585; learning_rate = 0.298; max_depth = 22; num_leaves = 4; min_child_samples = 7; subsample = 0.319; colsample_bytree = 0.930	0.0174	0.0132	0.0214	0.0192
	CatBoost	depth = 4; learning_rate = 0.106; l2_leaf_reg = 9.762	0.0135	0.0129	0.0185	0.0189

In the case of the *i*_*R*_/*i*_*Ro*_ output, the CatBoost model again demonstrated superior performance with the lowest test RMSE after tuning (0.0884), suggesting its robustness in handling this target. The XGBoost and LightGBM models followed closely, though all models experienced increased RMSE values post-tuning. This suggests that while tuning may not have reduced RMSE across the board, it likely improved the models’ generalization by preventing overfitting, especially for tree-based ensemble methods. For the *q*/*q*_*o*_ output, the CatBoost model continued to lead in both pre- and post-tuning scenarios, with the lowest test RMSE (0.0189 after tuning). The XGBoost and LightGBM models also delivered strong performance, with post-tuning test RMSEs of 0.0198 and 0.0192, respectively. The RF model, in contrast, showed a significant increase in test RMSE after tuning, which may point to overfitting or the need for better-tuned complexity constraints.

Across all three outputs, the CatBoost model consistently outperformed the RF and AdaBoost models in terms of predictive accuracy, particularly on the test data. Although tuning generally led to slightly higher training RMSEs, this effect is consistent with the goal of reducing overfitting and achieving better test set performance. The LightGBM and XGBoost model, with their flexible boosting frameworks and fine-tuned regularization, also demonstrated reliable performance gains through tuning. The AdaBoost and RF models, while simpler in nature, benefited less from the tuning process, highlighting the importance of model choice and complexity in hydraulic performance prediction tasks. Overall, the results reinforce the utility of hyperparameter tuning in enhancing model robustness and indicate that advanced gradient boosting algorithms like the CatBoost and LightGBM models are particularly well-suited for modeling hydraulic behavior with high precision.

### Cross-validation analysis

**Fig. 4:**
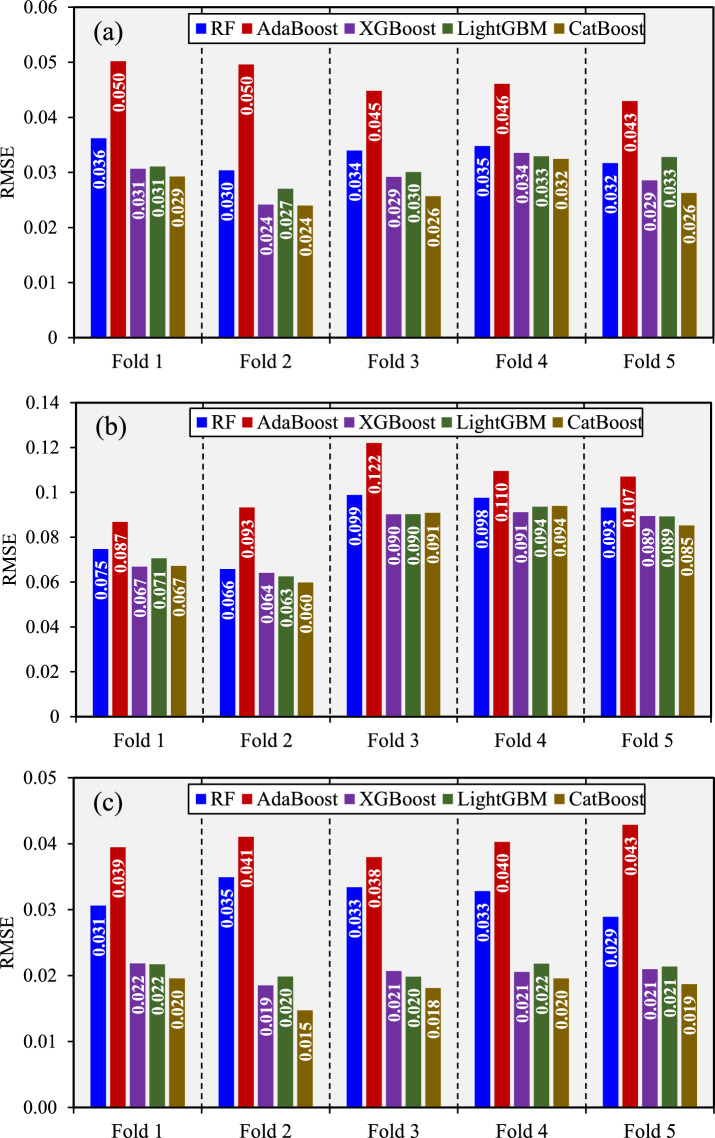
Comparison of model performance across five-folds CV process based on RMSE index (**a**) *U*/*U*_*o*_, (**b**) *i*_*R*_/*i*_*Ro*_, and (**c**) *q*/*q*_*o*_.

Figure 4 illustrates the performance of the applied models across five cross-validation folds using RMSE as the performance metric. This fold-wise analysis provides insight into each model’s stability and generalization capacity over different subsets of the data. In Fig. 4a, which corresponds to the *U*/*U*_*o*_ output, the CatBoost model consistently achieved the lowest RMSE values across nearly all folds, with scores ranging from 0.02398 to 0.03246. This indicates a high degree of model consistency and robustness. The XGBoost and LightGBM models also performed competitively, with the XGBoost model particularly excelling in Fold 2 (0.02415) and Fold 3 (0.02917). On the other hand, the RF and AdaBoost models showed higher RMSE values throughout all folds, with the AdaBoost model producing the highest error rates among all models, particularly in Fold 1 (0.05019) and Fold 2 (0.0496). The results suggest that boosting algorithms with more advanced regularization (CatBoost and XGBoost) outperform simpler ensemble methods for this output.

Figure 4b represents *i*_*R*_/*i*_*Ro*_, further emphasizes the CatBoost model’s consistency, with RMSE values staying within a relatively narrow range of 0.05981 to 0.0939. Although the margin between models is smaller here compared to *U*/*U*_*o*_, the CatBoost model still slightly outperforms its peers in most folds. The XGBoost and LightGBM models show very similar performance to each other, with the XGBoost model showing the lowest error in Fold 2 (0.06412). The RF model, while not the worst performer, demonstrates higher variability and slightly larger RMSEs, especially in Fold 3 (0.09885) and Fold 4 (0.09757). AdaBoost, once again, exhibits the highest RMSE across all folds, peaking in Fold 3 with a value of 0.122, which suggests that its simplistic learning strategy may struggle with the complexity of this particular target. In Fig. 4c, which addresses the *q*/*q*_*o*_ output, the superiority of the CatBoost model is once again apparent. It achieves the lowest RMSE in every fold, with values as low as 0.01471 in Fold 2 and consistently below 0.020 across the majority of the folds. The XGBoost model also delivers strong performance, staying close to the CatBoost model and showing its lowest RMSE at 0.01851 in Fold 2. The LightGBM model follows a similar trend with slight variations, maintaining a solid performance throughout the folds with RMSEs in the 0.019–0.021 range. Meanwhile, the RF and AdaBoost models trail significantly behind, particularly in Fold 2 and Fold 5, where the AdaBoost model reaches RMSEs of 0.04105 and 0.04287, respectively. Overall, Fig. 4 clearly demonstrates that the CatBoost model outperforms the other models across all outputs and folds in terms of predictive accuracy and consistency. The XGBoost and LightGBM models follow closely, making them strong contenders in scenarios where computational efficiency or interpretability is a higher priority. The RF and AdaBoost models, while still viable, consistently show weaker generalization.

### AIC analysis

Table 4 shows the performance of the adopted models based on the AIC value across three outputs. Lower AIC values indicate better model performance with respect to balancing goodness of fit and complexity. For *U*/*U*_*o*_, the CatBoost model achieves the lowest AIC value, suggesting it provides the best trade-off between accuracy and model simplicity, followed closely by the LightGBM and XGBoost models. For *i*_*R*_/*i*_*Ro*_, the CatBoost model again outperforms the other models, with a significantly lower AIC compared to the RF and AdaBoost models. For *q*/*q*_*o*_, the trend continues, with the CatBoost model displaying the lowest AIC, followed closely by the LightGBM and XGBoost models, both of which outperform the RF and AdaBoost models. This table indicates that, based on AIC, the CatBoost model consistently offers the most efficient performance across all outputs, while models like the XGBoost and LightGBM models also show strong performance, particularly in more complex outputs like *q*/*q*_*o*_.

**Table 4:** Models’ performance comparison based on the AIC value.

Output	RF	AdaBoost	XGBoost	LightGBM	CatBoost
*U*/*U*_*o*_	− 739.17	− 613.74	− 763.434	− 781.29	− 795.10
*i*_*R*_/*i*_*Ro*_	− 332.06	− 309.99	− 339.85	− 340.03	− 370.55
*q*/*q*_*o*_	− 733.45	− 703.68	− 937.06	− 942.52	− 953.66

### Model performance analysis

#### REC curves

**Fig. 5:**
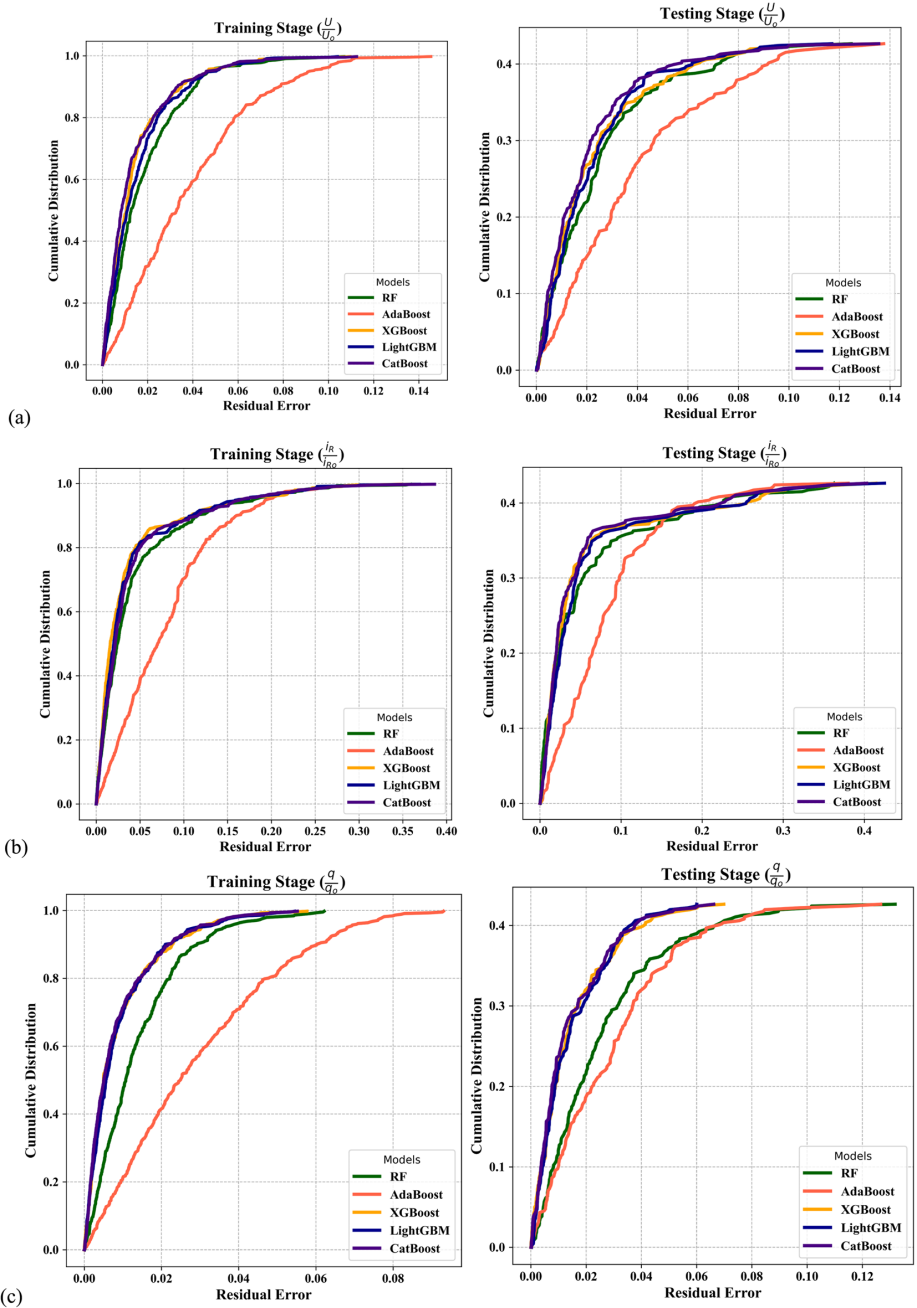
REC curves illustrating the performance of the adopted ML models during the training and testing phases for (**a**) *U*/*U*_*o*_, (**b**) *i*_*R*_/*i*_*Ro*_, and (**c**) *q*/*q*_*o*_.

Figure 5 presents the REC curves for the applied models during both training and testing stages for the three outputs. In Fig. 5a, the REC curves demonstrate the predictive accuracy of each model in estimating *U*/*U*_*o*_. The training stage shows high precision across all gradient boosting models, with the CatBoost and LightGBM models showing particularly steep curves indicating low residual error. In the testing phase, the CatBoost model continues to lead, achieving the highest cumulative distribution at lower error thresholds, confirming its strong generalization capability in predicting *U*/*U*_*o*_ ratio. Figure 5b illustrates the REC curves for *i*_*R*_/*i*_*Ro*_. During training, all models except the AdaBoost model show sharp convergence, indicating strong fit; however, the AdaBoost model shows higher residuals, reflecting poorer training accuracy. In testing, the CatBoost model again maintains superior performance, exhibiting the lowest spread in error and highest cumulative gain at minimal residual values, making it the best performer for this output as well.

Figure 5c shows the model performance for predicting *q*/*q*_*o*_ Also, the CatBoost and LightGBM models show excellent training performance with narrow error margins. In the testing stage, the CatBoost model delivers the steepest REC curve, followed closely by the LightGBM model, signifying the highest predictive reliability among the evaluated models. The RF model maintained moderately competitive performance across all outputs, often outperforming the AdaBoost model, particularly in the testing stages, but slightly lagging behind the gradient boosting variants in terms of both residual accuracy and generalization. Across all three outputs and both stages, the CatBoost model consistently demonstrated the best performance, offering the most accurate and stable predictions, particularly in generalizing to unseen data.

#### Scatter plots

**Fig. 6:**
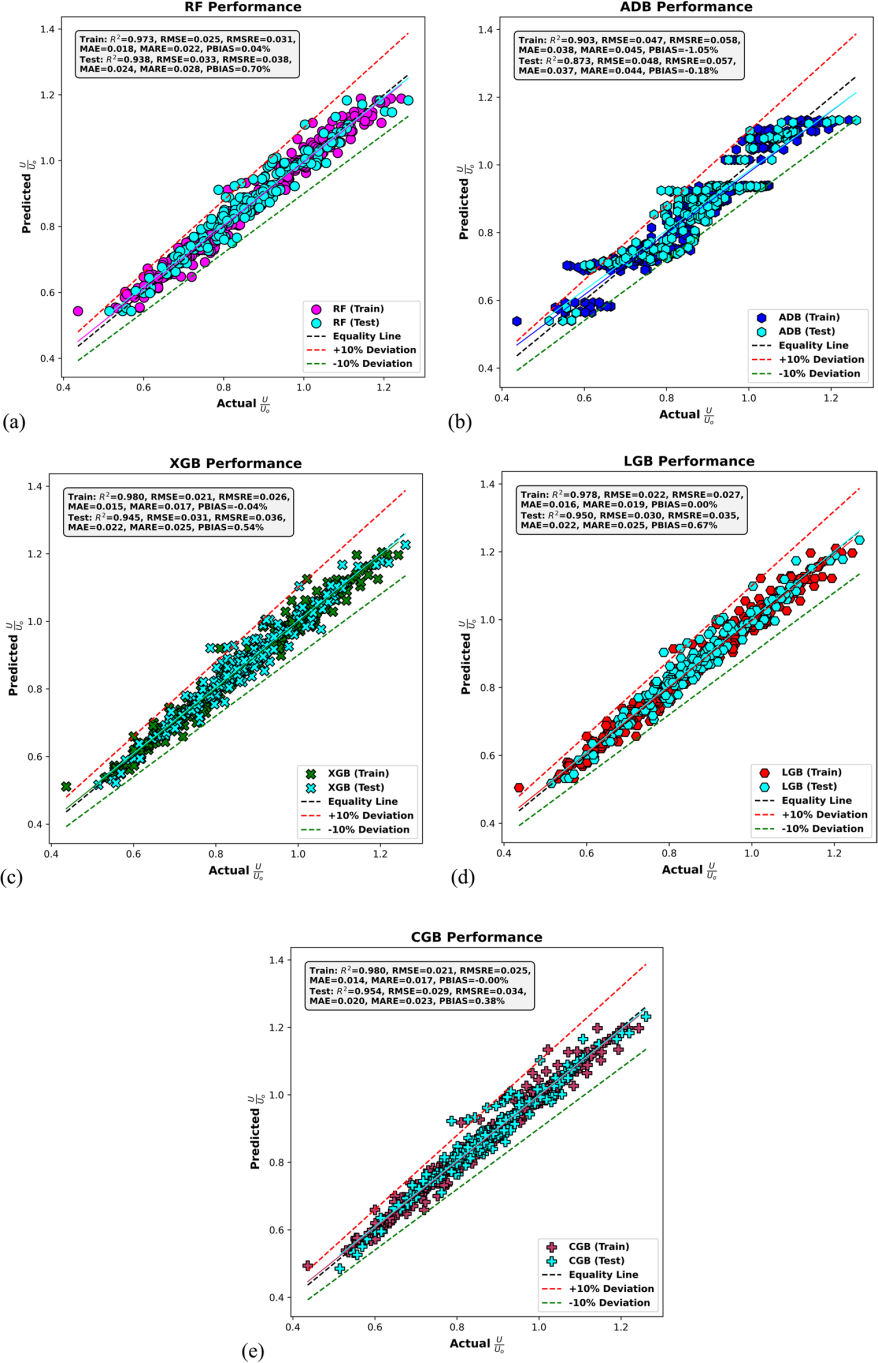
Actual versus predicted *U*/*U*_*o*_ values based on (**a**) RF, (**b**) AdaBoost (ADB), (**c**) XGBoost (XGB), (**d**) LightGBM (LGB), and (**e**) CatBoost (CGB) models in the training and testing stages.

Figure 6 illustrates scatter plots of actual versus predicted *U*/*U*_*o*_ values for the different ML models during both training and testing stages. Points clustering closely around the equality line indicate higher prediction accuracy, while the ± 10% deviation lines serve as visual references for acceptable model performance. In Fig. 6a, the RF model demonstrates strong predictive capability. During training, it achieves an R^2^ of 0.973, with low RMSE and MAE values of 0.025 and 0.018, respectively. The RMSRE and MARE are also low at 0.031 and 0.022, and the PBIAS is nearly negligible at 0.04%, indicating accurate and unbiased predictions. In the testing stage, RF maintains high performance with an R^2^ of 0.938, RMSE of 0.033, and MAE of 0.024. The RMSRE and MARE slightly increase to 0.038 and 0.028, respectively, while the PBIAS remains low at 0.70%, confirming good generalization with only a slight drop in accuracy.

Figure 6b shows the performance of the ADB model, which is comparatively weaker than the other models. In the training phase, the ADB model achieves an R^2^ of 0.903, but with higher RMSE and MAE values of 0.047 and 0.038, respectively. The RMSRE and MARE are 0.05 and 0.044, indicating larger relative errors. The PBIAS is − 1.05%, suggesting a slight underestimation. In testing, the performance further declines, with an R^2^ of 0.873, RMSE of 0.048, and MAE of 0.037. The RMSRE and MARE increase to 0.057 and 0.045, respectively, and the PBIAS is − 0.18%, reinforcing the model’s tendency to underestimate and its limited generalization ability. In Fig. 6c, the XGBoost (XGB) model demonstrates excellent predictive performance. The training stage yields an R^2^ of 0.980, with RMSE and MAE values of 0.021 and 0.015. The RMSRE and MARE are also low, at 0.026 and 0.017, and the PBIAS is minimal at− 0.04%. In the testing phase, the XGBoost model maintains strong performance with an R^2^ of 0.945, RMSE of 0.031, and MAE of 0.022. The RMSRE and MARE are 0.036 and 0.025, respectively, and the PBIAS remains low at 0.54%, indicating effective generalization and minimal bias. Figure 6d highlights the performance of the LightGBM (LGB) model, which also shows consistently strong results. In the training phase, the LGB model achieves an R^2^ of 0.978, with RMSE and MAE values of 0.022 and 0.016. The RMSRE and MARE are 0.027 and 0.019, and the PBIAS is effectively zero. During testing, the LGB model continues to perform well, with an R^2^ of 0.950, RMSE of 0.030, and MAE of 0.022. The RMSRE and MARE are 0.035 and 0.025, and the PBIAS is 0.67%, demonstrating both accuracy and stability across datasets.

Finally, Fig. 6e presents the CatBoost (CGB) model, which ranks among the top-performing models. In training, it achieves an R^2^ of 0.980, with very low RMSE and MAE values of 0.021 and 0.014. The RMSRE and MARE are 0.025 and 0.017, respectively, with a near-zero PBIAS of − 0.0003%, indicating highly accurate and unbiased predictions. In testing, the CatBoost model maintains excellent results with an R^2^ of 0.954, RMSE of 0.029, and MAE of 0.020. The RMSRE and MARE are 0.034 and 0.023, and the PBIAS is 0.38%, confirming strong generalization with minimal error. Overall, the results confirm that the CatBoost model, the XGBoost, and LightGBM models deliver superior performance with high accuracy, low error metrics, and strong generalization. The RF model also performs well, while AdaBoost shows relatively lower predictive accuracy, particularly in the testing stage.

**Fig. 7:**
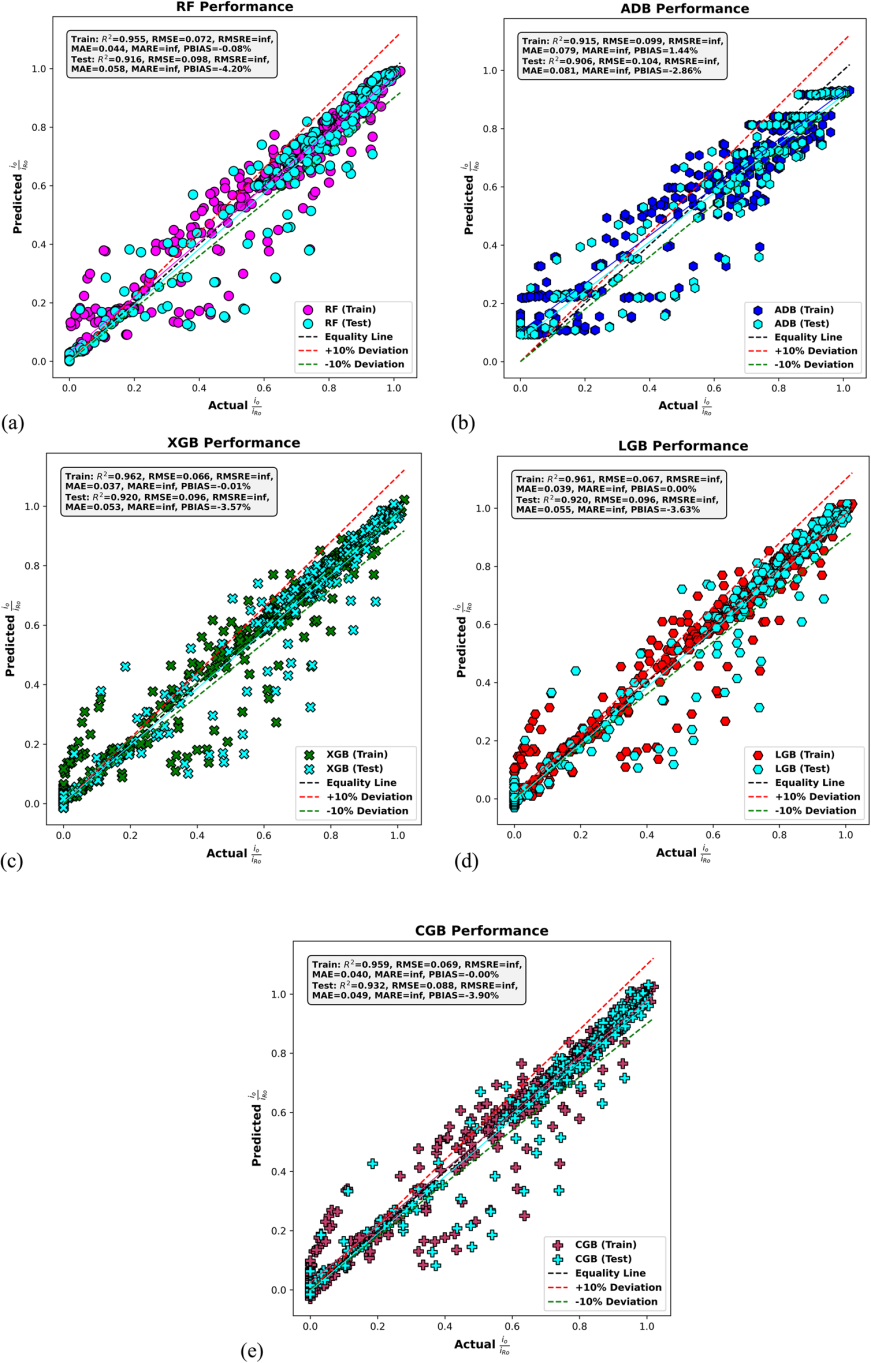
Actual versus predicted *i*_*R*_/*i*_*Ro*_ values based on (**a**) RF, (**b**) AdaBoost, (**c**) XGBoost, (**d**) LightGBM, and (**e**) CatBoost models in the training and testing stages.

Figure 7 shows scatter plots of predicted versus actual values for the second output variable *i*_*R*_/*​i*_*Ro*_​​ across training and testing stages for each model. Each subplot includes ± 10% deviation boundaries from the equality line to visually assess the prediction accuracy. In Fig. 7a, the RF model exhibits strong predictive alignment, with most data points clustered around the equality line. It achieves an R^2^ of 0.955 in training and 0.916 in testing, with RMSE values of 0.072 and 0.098, respectively. The MAE remains low at 0.044 for training and 0.058 for testing, while the PBIAS is minimal, at − 0.08% for training and − 4.20% for testing. The slight increase in residual spread during testing reflects a minor degradation in generalization. Figure 7b displays the performance of the ADB model, which shows the weakest predictive consistency among the models. While the training R^2^ is 0.915 and test R^2^ is 0.906, both stages suffer from higher RMSE (0.099 for training, 0.104 for testing) and MAE (0.079 and 0.081, respectively). The model also shows a positive PBIAS of 1.44% in training and a negative PBIAS of − 2.86% in testing, indicating a notable deviation from balanced predictions, particularly at extreme values.

Figure 7c presents the results for the XGB model, which demonstrates excellent predictive ability and generalization. The model records the highest training R^2^ at 0.962 and matches the LGB model in testing with an R^2^ of 0.920. The RMSE values are low (0.066) for training and 0.096 for testing, while the MAE stands at 0.037 and 0.053, respectively. The testing PBIAS of − 3.57% reflects a slight underprediction, but overall, the XGB model provides robust and balanced predictions. In Fig. 7d, the LGB model maintains strong consistency across both stages, with R^2^ values of 0.961 in training and 0.920 in testing, closely matching XGB. The RMSE is 0.067 and 0.096, while MAE values are 0.039 and 0.055, respectively. The PBIAS remains close to zero in training and only − 3.63% in testing, indicating reliable predictive performance with limited overfitting.

Finally, Fig. 7e shows the performance of the CGB model, which also delivers high prediction accuracy. The training and testing R^2^ values are 0.959 and 0.932, respectively, the highest testing score among all models. The RMSE values are 0.069 (train) and 0.088 (test), and MAE values are 0.040 and 0.049, with a very low prediction bias (− 0.0025% in training and − 3.90% in testing). The clustering of predicted values around the equality line within the ± 10% margin confirms the CatBoost model’s excellent generalization and minimal error variance. These plots highlight the superior performance of the CatBoost, XGBoost, and LightGBM models, with the CatBoost model achieving the best generalization in predicting *i*_*R*_/*​i*_*Ro*_​​, as evidenced by its high test R^2^, low error metrics, and minimal prediction bias.

**Fig. 8:**
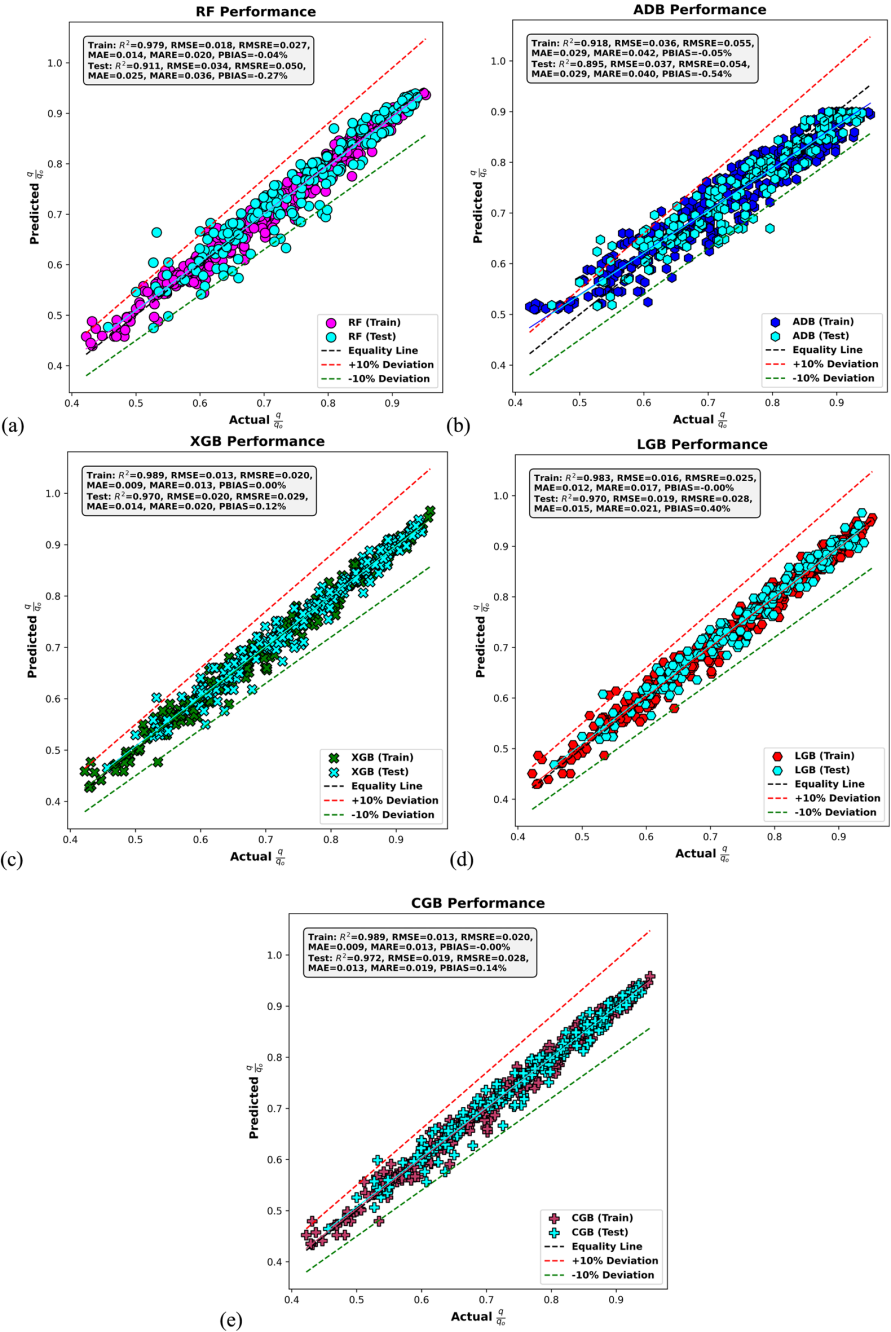
Actual versus predicted *q*/*q*_*o*_ values based on (**a**) RF, (**b**) AdaBoost, (**c**) XGBoost, (**d**) LightGBM, and (**e**) CatBoost models in the training and testing stages.

Figure 8 displays scatter plots of actual versus predicted values for *q*/*q*_*o*_ during both training and testing phases, across five machine learning models. Each subplot includes an equality line and ± 10% deviation boundaries to assess model accuracy. Figure 8a illustrates the performance of the RF model, which performs well, particularly in the training phase with an R^2^ of 0.979 and low RMSE of 0.018. However, in testing, performance declines slightly to an R^2^ of 0.911 and RMSE of 0.034, with a PBIAS of − 0.27%, suggesting mild underprediction. Nevertheless, the majority of the predictions remain within the ± 10% deviation bounds. Figure 8b shows the ADB performance, which records lower predictive performance relative to the other models. The training R^2^ is 0.918 and testing R^2^ is 0.895, both among the lowest. Error metrics such as RMSE (0.036 for training and 0.037 for testing) and MAE (0.029 in both stages) remain significantly higher than those of the top-performing models. The higher PBIAS in testing (− 0.54%) further indicates a tendency toward underprediction, especially at mid-range values. In Fig. 8c, the XGB model achieves the best overall performance. With an R^2^ of 0.989 in training and 0.970 in testing, the model demonstrates exceptional accuracy and generalization. The RMSE is remarkably low as 0.013 for training and 0.020 for testing, while MAE values are also minimal at 0.009 and 0.014, respectively. The model’s PBIAS is negligible, at 0.00% for training and 0.12% for testing, confirming a strong alignment with the equality line and consistency across the dataset.

Figure 8d presents the LGB model, which also shows strong predictive performance. It achieves an R^2^ of 0.983 for training and 0.970 for testing, closely aligning with the XGBoost and CatBoost models. RMSE values are low as 0.016 in training and 0.019 in testing, while MAE values are 0.012 and 0.015, respectively. A slight positive prediction bias is noted in testing (0.40%), but overall, predictions remain tightly clustered around the equality line. Figure 8e highlights the CGB model, which performs on par with XGBoost. It achieves an R^2^ of 0.989 in training and 0.972 in testing, the highest among all models for this output. The RMSE values are 0.013 and 0.019, and the MAE values are similarly low at 0.009 and 0.013 for training and testing, respectively. The model exhibits nearly zero bias (PBIAS ≈ 0%) in both stages, reflecting excellent stability and predictive power. Overall, the CatBoost and XGBoost models outperform all other models in predicting *q*/*q*_*o*_, showing the lowest residual errors, minimal bias, and highest R^2^ values in both training and testing phases, confirming their superior modeling ability.

### Violin boxplots

**Fig. 9:**
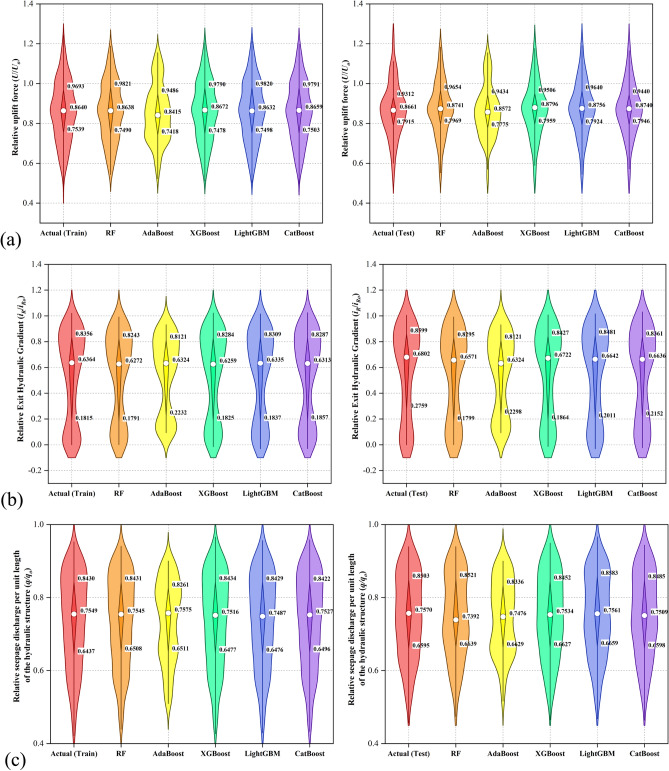
Violin boxplots depicting the distribution of actual and predicted values across the training and testing stages for (**a**) *U*/*U*_*o*_, (**b**) *i*_*R*_/*i*_*Ro*_, and (**c**) *q*/*q*_*o*_.

Figure 9 presents violin boxplots comparing the distribution of actual and predicted values across both training and testing datasets for three outputs. For the *U*/*U*_*o*_, all gradient boosting models produce prediction distributions closely aligned with the actual data, showing good symmetry and overlapping interquartile ranges in both training and testing stages. Among them, the CatBoost and LightGBM models exhibit the narrowest distributions and highest median alignment, indicating strong accuracy and low variability. The AdaBoost model shows slightly more spread, suggesting weaker precision. For *i*_*R*_/*i*_*Ro*_, the predicted distributions by all models tend to be narrower than the actual distribution, especially in the test set. This may indicate a slight underestimation of variability. The CatBoost and LightGBM models again demonstrate superior performance with distributions closely capturing both the central tendency and spread of the actual values. The AdaBoost model shows more bias toward lower values, especially in the testing stage. For *q*/*q*_*o*_, all models achieve strong alignment with the actual distributions, particularly in the central region. The XGBoost and CatBoost models provide the most accurate approximations, maintaining consistent median and quartile values across both training and testing phases. AdaBoost again shows a slight tendency to underpredict in some regions, as indicated by narrower plots and a lower lower-bound whisker. Overall, the violin plots reinforce the superior performance of the CatBoost, LightGBM, and XGBoost models in accurately capturing not only central tendencies but also the full distribution of each output variable, thereby supporting their reliability for predicting the outputs.

### Taylor diagrams

**Fig. 10:**
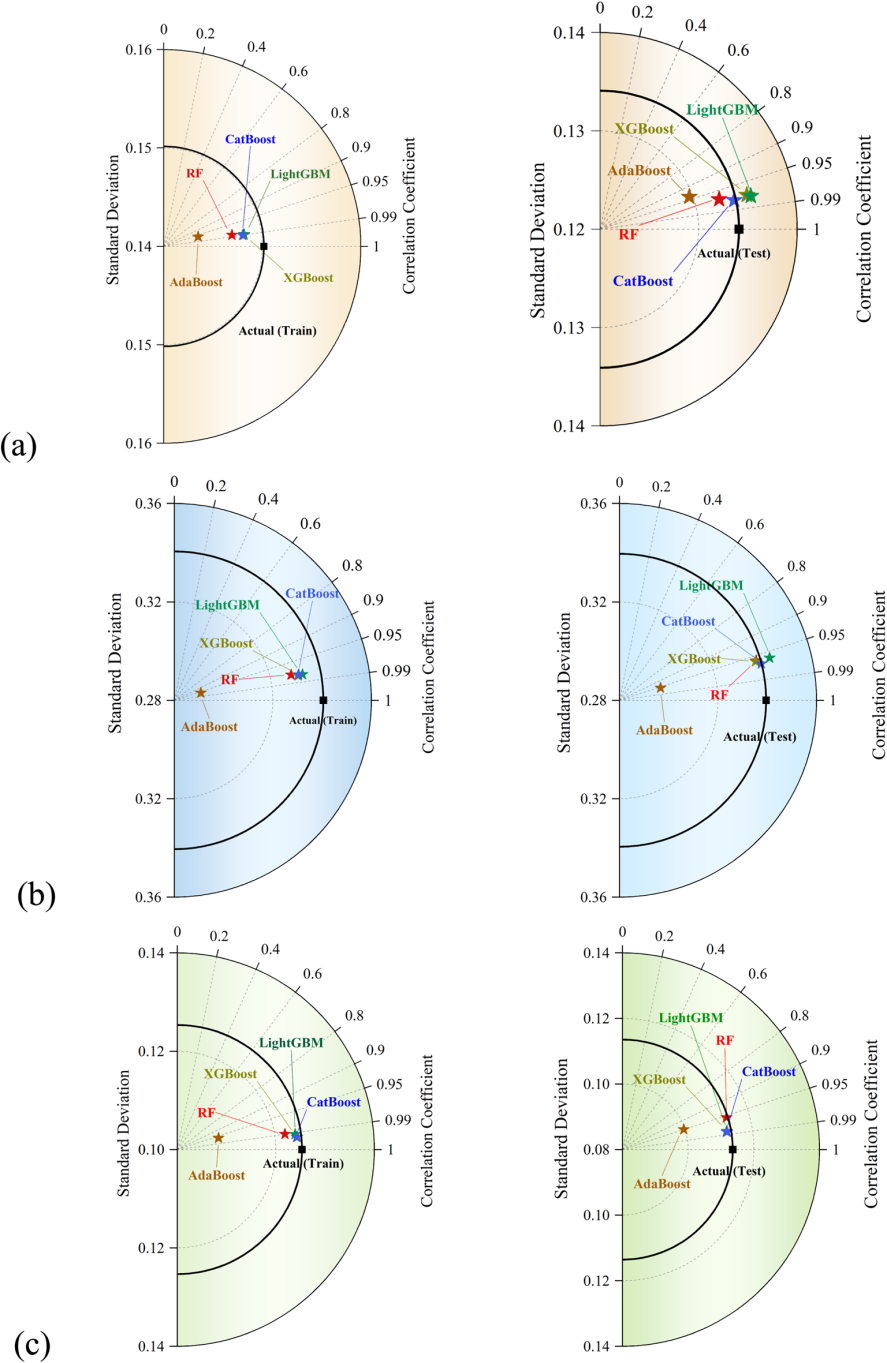
Taylor diagrams illustrating the agreement between actual and predicted values across the training and testing stages for (**a**) *U*/*U*_*o*_, (**b**) *i*_*R*_/*i*_*Ro*_, and (**c**) *q*/*q*_*o*_.

Figure 10 illustrates Taylor diagrams comparing the performance of the five applied machine learning models in both training and testing stages across the three outputs. For *U*/*U*_*o*_, the Taylor diagrams show that the CatBoost and LightGBM models consistently yield the highest correlation coefficients (close to 1) and lowest standard deviations, tightly clustered near the reference (actual) point in both training and testing phases. This confirms their superior predictive consistency and precision. The XGBoost model also performs well, though slightly further from the ideal point than the CatBoost model. AdaBoost model, on the other hand, exhibits the lowest correlation and higher deviation, especially in the test stage, reflecting its weaker generalization. For *i*_*R*_/*i*_*Ro*_​​, similar trends are observed. The CatBoost, LightGBM, and XGBoost models maintain high correlations and relatively small standard deviations, positioning them closest to the actual values. RF performs moderately well, while the AdaBoost model again shows the poorest alignment with the actual data distribution. Regarding *q*/*q*_*o*_, the Taylor diagrams reveal a slight increase in standard deviation across all models, particularly in the training phase. However, the CatBoost and XGBoost models still maintain high correlation values near the ideal point, demonstrating robustness. The LightGBM model also performs reliably with minimal overfitting signs. The RF model shows more deviation and lower correlation, while the AdaBoost model again appears furthest from the target point. Overall, across all outputs and stages, the CatBoost model consistently aligns most closely with the actual data, indicating its superior accuracy and generalization capability among the tested models.

### Uncertainty analysis

Table 5 shows the performance of the adopted models based on the U_95_ value for uncertainty analysis across training and testing datasets for three outputs. The U_95_ value represents the upper 95% confidence interval for prediction uncertainty, providing insights into the stability and reliability of the models.

**Table 5:** Performance of the adopted ML models based on U_95_ value for uncertainty analysis.

Output	Stage	RF	AdaBoost	XGBoost	LightGBM	CatBoost
*U*/*U*_*o*_	Train	0.069	0.115	0.059	0.062	0.059
	Test	0.092	0.128	0.086	0.082	0.079
*i*_*R*_/*i*_*Ro*_	Train	0.200	0.274	0.184	0.187	0.191
	Test	0.267	0.286	0.263	0.263	0.241
*q*/*q*_*o*_	Train	0.049	0.099	0.036	0.045	0.036
	Test	0.094	0.101	0.055	0.054	0.052

For *U*/*U*_*o*_, the uncertainty in the test set is lowest for the CatBoost model, followed closely by the XGBoost and LightGBM models, indicating these models offer more consistent predictions with less uncertainty. For *i*_*R*_/*i*_*Ro*_, all models exhibit higher uncertainty compared to *U*/*U*_*o*_ and *q*/*q*_*o*_, with the CatBoost model again demonstrating the lowest U_95_ value in the test set, reflecting better stability. For *q*/*q*_*o*_, the XGBoost and CatBoost models show the lowest uncertainty in both training and testing phases, suggesting they are more reliable for this output. Overall, the CatBoost and XGBoost models consistently exhibit lower uncertainty across outputs, implying they provide more stable and confident predictions, especially when tested on unseen data.

### Input feature ranking

**Fig. 11:**
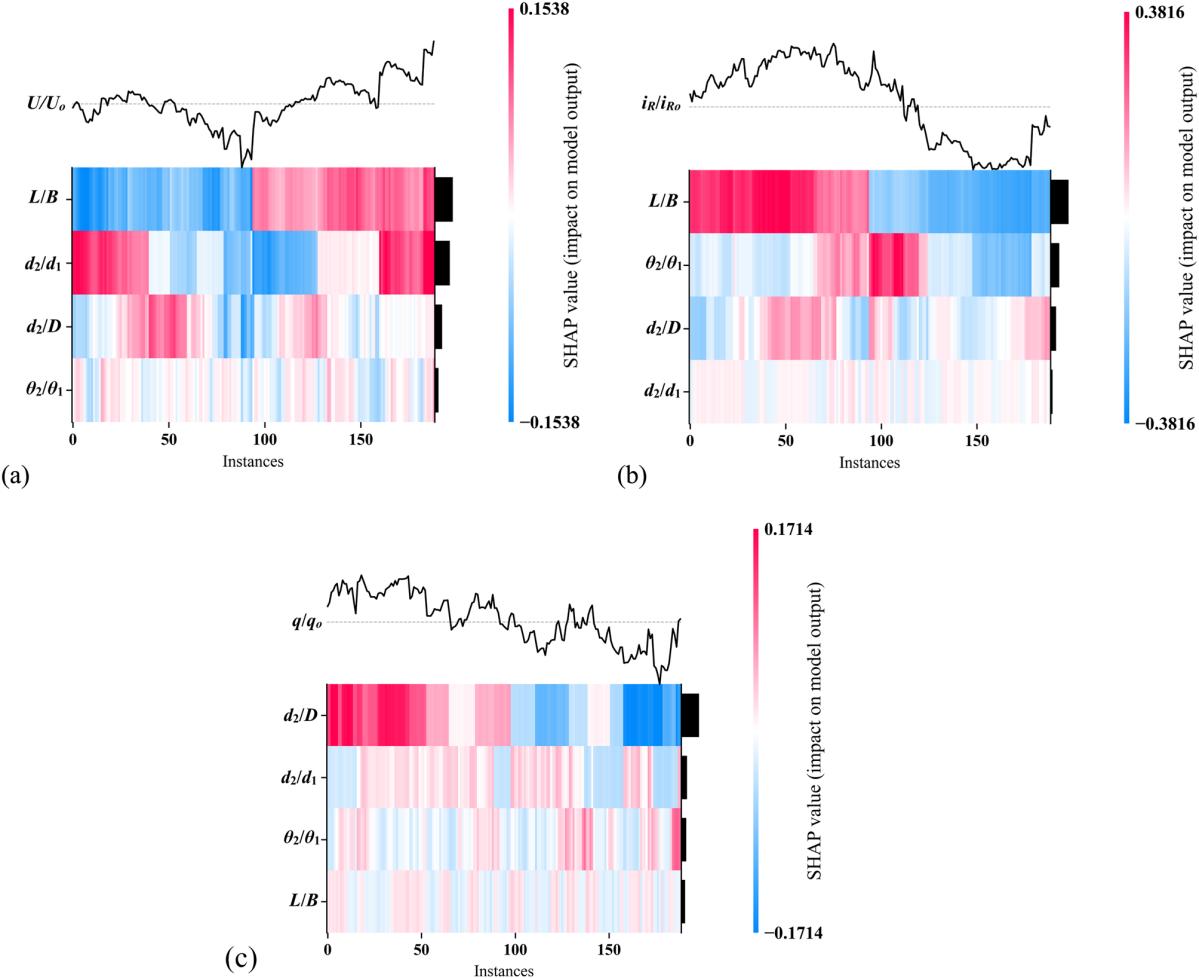
SHAP feature importance analysis heatmaps for (**a**) *U*/*U*_*o*_, (**b**) *i*_*R*_/*i*_*Ro*_, and (**c**) *q*/*q*_*o*_.

Figure 11 shows the SHAP heatmaps indicating feature rankings are shown for the three outputs based on the best predictor in the testing stage. Each plot illustrates how different features impact the model’s output, with color intensity representing the magnitude and direction of the SHAP values. Red indicates a positive contribution to the output, while blue indicates a negative contribution. In Fig. 11a, the heatmap for *U*/*U*_*o*_ shows that *L*/*B* having the most significant influence on the model’s output, with large areas of red and blue indicating that *L*/*B* contributes both positively and negatively depending on the instance. *d*_2_/*D* also shows a moderate impact with noticeable shifts in SHAP values, while *θ*_2_/*θ*_1_ and *d*_2_/*d*_1_ appear to have less influence across instances. Figure 11b, representing *i*_*R*_/*i*_*Ro*_, highlights *L*/*B* and *d*_2_/*d*_1_ as the most influential features. *L*/*B* contributes significantly both positively and negatively across multiple instances, as seen from the strong red and blue bands. *d*_2_/*d*_1_ displays consistent contributions, though slightly less intense. *θ*_2_/*θ*_1_ and *d*_2_/*D* show comparatively smaller impacts, with fewer and less intense color shifts throughout the instances. In Fig. 11c, the heatmap for *q*/*q*_*o*_ shows *d*_2_/*D* as the dominant feature, contributing the most to the output, as evidenced by the strong presence of red and blue across instances. *d*_2_/*d*_1_ also has a substantial impact but with less intensity. *θ*_2_/*θ*_1_ and *L*/*B* show relatively lower importance, with fewer marked contributions across the instances, as indicated by the lighter color patterns.

**Fig. 12:**
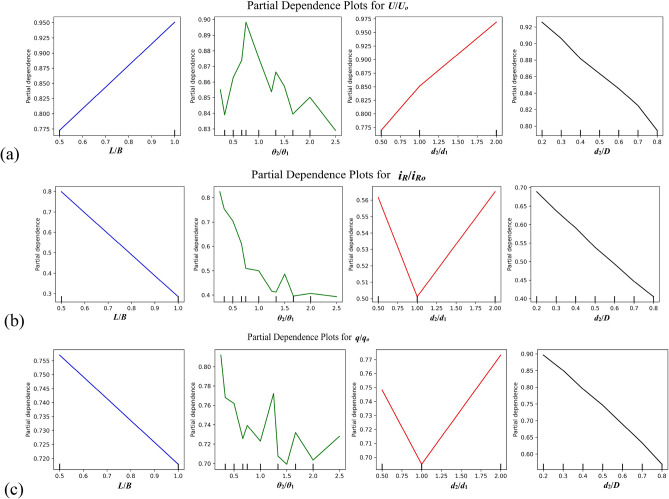
PDPs of input features interpretability for predicting (**a**) *U*/*U*_*o*_, (**b**) *i*_*R*_/*i*_*R*__*o*_, and (**c**) *q*/*q*_*o*_.

The analysis of PDPs reveals consistent patterns in the relationships between the four inputs and the predicted outcomes. In Fig. 12a, for *U*/*U*_*o*_, a positive linear relationship emerges between *L*/*B* and the output, while *θ*_2_/*θ*_1_ exhibits non-linear fluctuations. *d*_2_/*d*_1_ steadily increases its impact, and *d*_2_/*D* maintains a negative linear trend. In Fig. 12b, for *i*_*R*_/*i*_*Ro*_, *L*/*B* shows a negative linear relationship, *θ*_2_/*θ*_1_ displays non-linear fluctuations, and *d*_2_/*d*_1_ presents a V-shaped pattern. *d*_2_/*D* maintains a steady negative linear relationship. In Fig. 12c, *q*/*q*_*o*_ follows a similar pattern to *i*_*R*_/*i*_*R*__*o*_, with *L*/*B* showing a negative linear relationship, *θ*_2_/*θ*_1_ revealing a fluctuating non-linear trend, and *d*_2_/*d*_1_ displaying a V-shaped curve. *d*_2_/*D* maintains a steady negative linear impact across all three outcomes, with varying degrees of influence from *L*/*B*, *θ*_2_/*θ*_1_, and *d*_2_/*d*_1_.

### Graphical user interface

**Fig. 13:**
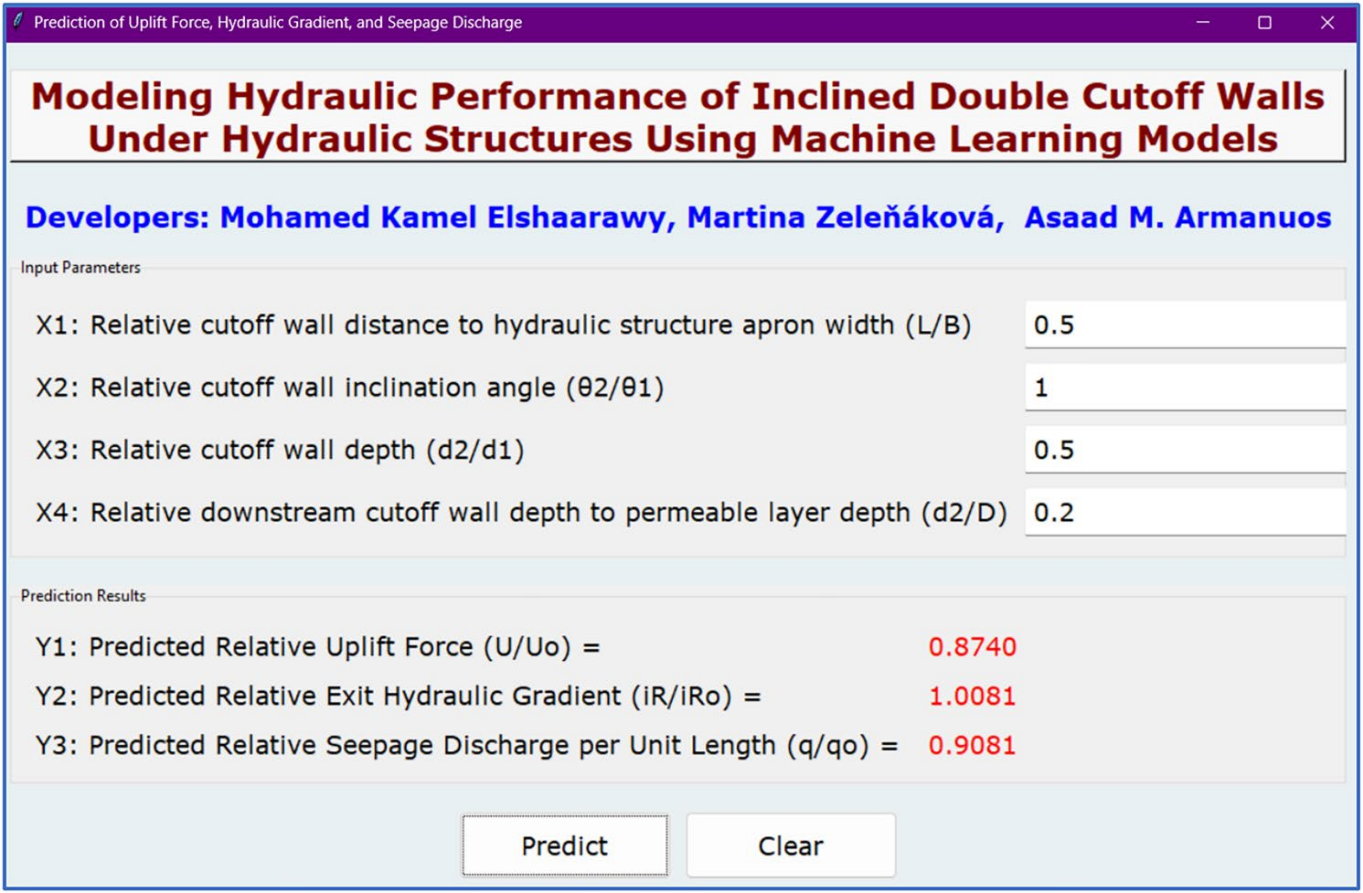
Screenshot of the developed GUI model for predicting the three outputs.

The GUI model, shown in Fig. 13, represents a significant advancement in applying machine learning to engineering design, specifically for predicting key outputs related to inclined double cutoff walls under hydraulic structures. It was built using the Tkinter package^78^ as a Python-based application allows users to input parameters such as relative cutoff wall distance, inclination angle, and wall depth to instantly calculate outputs like uplift force, exit hydraulic gradient, and seepage discharge. The tool, hosted on GitHub for easy access and collaboration, democratizes the use of advanced predictive models and invites further community-driven enhancements. Users can access the GUI at https://github.com/mkamel24/DCW.

## Discussion

### Model performance comparison

The comparative analysis of all adopted machine learning models clearly highlights the superior performance of the CGB model, followed closely by the XGB and LGB models, across all evaluation metrics and outputs. The combination of low RMSE, high R^2^ values, favorable AIC scores, and minimal uncertainty (U_95_) consistently places the CGB model at the forefront. Its ability to handle categorical variables natively and reduce overfitting through symmetric tree boosting likely contributed to its robustness. In contrast, the ADB model persistently exhibited the weakest predictive performance, with higher residual errors, flatter REC curves, and broader value distributions in both training and testing phases. This underperformance suggests that the ADB model may be less suitable for modeling complex, non-linear relationships present in this dataset.

### Effectiveness of Bayesian optimization

The BO process proved instrumental in refining hyperparameter settings and improving model generalization. While there was a modest increase in training RMSE for some models post-tuning, this was generally accompanied by improved or more stable test performance, indicating successful mitigation of overfitting. The tuning particularly enhanced performance in complex models such as the XGB and LGB models, ensuring they maintained a good balance between accuracy and model complexity. Furthermore, the optimized hyperparameters displayed considerable variability across outputs, affirming the need for output-specific tuning rather than a one-size-fits-all approach.

### Model efficiency and computational cost

This study incorporates the AIC as a statistical tool to reflect the trade-off between model complexity and goodness of fit. While AIC does not directly measure computational time, it provides valuable insight into the relative efficiency of models by penalizing those with excessive complexity. A lower AIC value suggests a model that achieves high accuracy without overfitting, while also avoiding unnecessary computational overhead. The AIC values presented in Table 5 indicate that CatBoost consistently outperforms the other models across all outputs, achieving the lowest AIC values. This indicates that CatBoost provides the best balance between accuracy and model simplicity.

In the case of *U*/*U*_*o*_, the CatBoost model delivers the most efficient performance, followed closely by the LightGBM and XGBoost models. These models are particularly well-suited for tasks that require both high predictive power and scalability, as reflected by their strong AIC performance. For *i*_*R*_/*i*_*R*__*o*_ and *q*/*q*_*o*_, the CatBoost model again leads the way, with significantly lower AIC values compared to other models, particularly when compared to the RF and AdaBoost models. While models like the AdaBoost and RF models are simpler and generally faster to train, their higher AIC scores indicate reduced efficiency and less desirable predictive performance, especially in more complex outputs like *q*/*q*_*o*_.

### Model interpretability insights

The integration of SHAP and PDP analyses provided vital interpretability into the behavior of the models and the influence of each input feature. Notably, feature importance varied significantly depending on the output variable. For instance, *L*/*B* was the most impactful for *U*/*U*_*o*_ and *i*_*R*_/*i*_*R*__*o*_, while *d*_2_/*D* dominated *q*/*q*_*o*_. This variability underscores the importance of feature-target dependency and the value of model interpretability tools in understanding complex relationships. The PDP plots confirmed and expanded upon the SHAP findings, revealing both linear and non-linear patterns, including V-shaped responses and fluctuating behaviors that suggest interaction effects and thresholds in the input–output relationships.

### Practical implications and application through GUI

The development of a GUI marks a pivotal step toward real-world applicability of the research. By translating complex ML models into an accessible, user-friendly tool, the system empowers engineers and practitioners to predict uplift force, exit gradient, and seepage discharge with high precision and minimal technical overhead. The open-source availability of the GUI further fosters transparency, reproducibility, and collaborative development. This bridge between sophisticated ML techniques and practical engineering applications exemplifies the potential of data-driven tools in modern geotechnical and hydraulic design practices.

## Conclusions

This study introduces a novel approach to predicting key hydraulic parameters in structures with inclined double cutoff walls using advanced ML models. It overcomes the limitations of traditional analytical methods by leveraging the CatBoost, XGBoost, and LightGBM models, achieving higher accuracy in forecasting uplift force, exit hydraulic gradient, and seepage discharge. The BO method enhances model performance, while the SHAP and PDPs provide insights into variable influences. Additionally, a user-friendly GUI bridges the gap between computational models and practical hydraulic engineering applications, making these tools more accessible for real-world use. Summing up the results, the following conclusions can be drawn:

- The CatBoost, LightGBM, and XGBoost models consistently outperformed traditional models like the RF and AdaBoost models in predicting uplift force, exit hydraulic gradient, and seepage discharge. The CatBoost model emerged as the most accurate and reliable model across all outputs.
- The study demonstrated the significance of tailored hyperparameter tuning using BO method. Proper tuning significantly improved model performance, particularly in reducing overfitting and improving generalization across test datasets.
- The research established a thorough evaluation process using regression metrics, AIC, REC curves, scatter plots, Taylor diagrams, and uncertainty analysis, ensuring that model performance was accurately assessed.
- The SHAP and PDP analyses offered valuable insights into the impact of key features such as depth, inclination angle, and cutoff wall distance on the predicted hydraulic parameters. This enhanced the understanding of feature importance, aiding in the optimization of hydraulic structure designs.
- The developed GUI based on the predictive models made the research findings more accessible to practitioners, facilitating the practical application of these advanced models in water resource management.

While this study offers valuable insights into the predictive performance of machine learning models for hydraulic structures, several limitations need to be addressed. The models were tested on static datasets, which may not accurately reflect the dynamic, time-varying conditions found in real-world scenarios. Additionally, the study focused on a narrow range of input parameters, limiting the applicability of the models to other hydraulic structures. Future research should focus on incorporating dynamic scenarios and time-series data to better capture real-world variability. Exploring advanced techniques such as deep learning models and hybrid methods could improve prediction accuracy further. Additionally, evaluating computational costs by measuring training time, memory usage, and inference speed will ensure a comprehensive assessment of model efficiency for practical engineering applications.

## Data Availability

Data, models, or codes that support the findings of this study are available from the corresponding author upon reasonable request.
